# Epidemiology, evolution, and biological characteristics of H6 avian influenza viruses in China

**DOI:** 10.1080/22221751.2022.2151380

**Published:** 2022-12-20

**Authors:** Xiaohao Xu, Qi Chen, Min Tan, Jia Liu, Xiyan Li, Lei Yang, Yuelong Shu, Dayan Wang, Wenfei Zhu

**Affiliations:** a School of Public Health (Shenzhen), Sun Yat-sen University, Guangdong, People’s Republic of China; b National Institute for Viral Disease Control and Prevention, Chinese Center for Disease Control and Prevention; WHO Collaborating Center for Reference and Research on Influenza; Key Laboratory for Medical Virology, National Health and Family Planning Commission, Beijing, People’s Republic of China; c Institute of Pathogen Biology, Chinese Academy of Medical Sciences & Peking Union Medical College, Beijing, People’s Republic of China

**Keywords:** H6 avian influenza viruses, epidemiology, evolution, pathogenicity

## Abstract

H6 avian influenza virus (AIV) is one of the most prevalent AIV subtypes in birds globally. To investigate the current situation and characteristics of H6 AIVs circulating in China, we analysed the epidemiology, genetic evolution and pathogenic features of this subtype. During 2000-2021, H6 subtype AIVs spread widely through Southern China and presented high host diversity. On analysing 171 H6 viruses isolated during 2009-2021, dynamic reassortments were observed among H6 and other co-circulating AIV subtypes, and these generated a total of 16 different genotypes. A few H6N6 strains possessed L226 and S228 mutations of hemagglutinin (H3 numbering), which may enhance the affinity of H6 viruses to human receptors. H6N6 viruses also exhibited divergent pathogenicity and growth profiles *in vivo* and *in vitro*. Some of the H6N6 viruses could infect mice without mammalian adaptation, and even caused death in this species. Therefore, our study demonstrated that the H6 AIVs posed a potential threat to human health and highlighted the urgent need for continued surveillance and evaluation of the H6 influenza viruses circulating in the field.

## Introduction

During the last century, four influenza pandemics were documented: H1N1 in 1918, H2N2 in 1957, H3N2 in 1968 and H1N1 influenza in 2009 [1]. Each pandemic resulted in significant mortality and morbidity numbers [2].

The genome segments of pandemic viruses were partially derived from avian influenza viruses (AIVs) [3]. Over the last few decades, an increasing number of outbreaks of highly pathogenic avian influenza viruses in poultry and wild birds have been reported. Several AIV subtypes currently circulating in poultry, in particular H5, H7 and H9, have crossed the species barrier and have infected and killed mammals, including humans, and thus have presented a major public health threat [4,5].

Subtype H6 low pathogenic avian influenza viruses are currently circulating widely throughout the globe. In 1965, H6 AIVs were isolated from a turkey in Massachusetts in the United States [6], and since then, H6 viruses have been sequentially reported in Chinese Taipei in 1972; Australia, the United States, and Canada in and around 1974; and Hong Kong in 1977 [7]. The isolation rate of H6 viruses from wild, domestic aquatic and terrestrial avian species throughout the world is increasing [6]. H6 AIVs are currently one of the most commonly detected subtypes in domestic ducks in Southern China, and have an extensive range of hosts [8,9]. H6 AIVs generally cause asymptomatic infections in waterfowl; however, infected chickens tend to display decreased egg production, upper respiratory tract infection symptoms, and high morbidity and mortality [8,10–13]. Previous studies have indicated that some H6 viruses can infect and cause illness in mice and ferrets without preadaptation [14,15]. In Southern China, H6N6 AIVs were isolated from swine with symptoms. Importantly, H6N1 virus was isolated from a 20-year-old woman with flu-like symptoms including cough, fever, headache, muscle ache, in May 2013 in Taiwan [15,16], highlighting the increasing potential threat of H6 subtype viruses to public health.

To monitor AIV genetic evolution and evaluate AIV pandemic potential in a timely manner, a sustained surveillance of live poultry markets (LPMs) has been conducted in mainland China since 2009. In this study, we investigated characteristics of the epidemiology, genetic evolution and pathogenicity of H6 AIVs in China.

## Materials and methods

### Ethics statement

Mouse experiments were carried out with the approval of the Ethics Committee of the National Institute for Viral Disease Control and Prevention, China CDC (20211119087). All experiments associated with live H6 viruses were conducted in biosafety level 2 (BSL2) laboratories.

### Virus isolation and titration

In this study, the H6 AIVs were isolated from environmental samples from LPMs or poultry farms according to the guideline on AIV surveillance of Chinese Centre for Disease Control and Prevention (China CDC). Samples were collected from avian-linked environments once per-month across 31 provinces, autonomous regions, and municipalities in mainland China. Viruses were isolated in specific pathogen free (SPF) embryonated chicken eggs at 37°C for 48 hours. A hemagglutinin (HA) assay was run with 1% turkey red blood cells (TRBCs) to confirm the presence of influenza viruses in the allantoic fluid of the eggs. The HA subtypes of all viruses were identified by real-time reverse transcription-PCR (real-time RT–PCR). Virus titrations were conducted in 9-day-old SPF embryonated eggs to determine the 50% embryo infectious dose (EID_50_) with the Reed and Muench method [17].

### Epidemiology analysis

From January 2009 to August 2021, a total of 171 H6 subtype AIV (18 H6N2 viruses, 151 H6N6 viruses and 2 H6N8 viruses) were isolated from live poultry related environmental samples by the Chinese National Influenza Centre (CNIC). In order to study the epidemiology of H6 subtype viruses in China, a total of 1524 H6 sequences, generated by the viruses from 2000 to 2021, were downloaded from the public databases, including the Global Initiative of Sharing All Influenza Database (GISAID), GenBank (National Centre for Biotechnology Information, NCBI), and CNIC. Information on the subtypes, hosts and geographic distributions of these 1524 H6Nx subtype viruses was collected for further spatiotemporal analysis.

### Whole-genome sequencing and phylogenetic analysis

An RNeasy Mini kit (Qiagen, Hilden, Germany) was used to extract virus RNA from the allantoic fluid of infected SPF embryonated eggs. The complete genome of H6 AIV was characterized by Illumina next-generation sequencing as follows. The extracted RNA was subjected to reverse transcription and amplification. Then whole-genome sequencing of FluA on the Miniseq high-throughput sequencing platform was implemented [18]. Data analysis and genome sequence acquisition were conducted using an established pipeline in our laboratory. After that, Velvet version 1.2.10 (https://guix.gnu.org/en/packages/velvet-1.2.10External Link) and Newbler Assembler version 2.5 were used to trim low-quality reads, and sample and de novo assemble the filtered reads. Then, all contigs were blasted against a database generated by CD-HIT which clustered all FluA sequences collected from the NCBI Influenza Virus Database and GISAID EpiFlu database (http://www.gisaid.orgExternal Link). After that, sequences with the highest similarity were selected as references and bowtie2 version 2.1.0 (https://sourceforge.net/projects/bowtie-bio/files/bowtie2/2.1.0External Link) was used for read mapping. Lastly, FluA genome sequences were obtained by extracting the consensus sequences from the mapping results, with a coverage depth of at least 30× at each site on the eight segments.

To build the system phylogeny tree by BEAST v1.10.4 (https://beast.community/2018-11-14.10.4_released.html), all sequences were aligned using MAFFT (version 7.222; http://mafft.cbrc.jp/alignment/software/). By utilizing the model of nucleotide substitution selected in Model Finder of IQ-TREE (version 2.1.1; http://www.iqtree.org/), phylogenetic trees were generated with a Bayesian Markov Chain Monte Carlo method. Best-fit clock model (the uncorrelated relaxed clock) was selected using marginal likelihoods. The Markov Chain Monte Carlo (MCMC) analyses were run for 100 million generations, with samples drawn every 10,000 generations. Lastly, phylogenetic trees were visualized in iTOL (https://itol.embl.de/) and Figtree (version 1.4.4; http://tree.bio.ed.ac.uk/software/figtree/).

### Virus growth kinetics study

Madin–Darby canine kidney cells (MDCK) were maintained in Dulbecco’s Modified Eagle’s Medium (DMEM, Invitrogen, Carlsbad, CA, USA) supplemented with 10% foetal bovine serum (FBS, Invitrogen), HEPES (10 mM, Invitrogen), penicillin (100 units/mL, Invitrogen), and streptomycin (100 µg/mL, Invitrogen). Confluent MDCK cells were infected with selected viruses at the multiplicity of infection (MOI) of 0.001. After 1 hour of adsorption, the viral inoculums were removed, then the inoculated cells were incubated at 37°C with DMEM containing 2 μg/mL TPCK-treated trypsin (Sigma-Aldrich, St Louis, MO, USA). Supernatants were collected at 0-, 6-, 12-, 24-, 36-, 48-, 60- and 72- hours post infection (hpi), respectively, and were stored at-80°C before viral titration in MDCK cells. The tissue culture infectious dose affecting 50% of the cells (TCID_50_) was calculated using the Reed–Muench formula [19].

### Neuraminidase inhibition assay

Oseltamivir carboxylate (oseltamivir; Hoffman-La Roche) was prepared in sterile distilled water and stored at -20 °C. Neuraminidase (NA) inhibitor (NAI) susceptibility was assessed by a fluorescence-based NA enzyme inhibition assay based on the NA-Fluor™ kit (Applied Biosystems, Foster City, USA). The IC_50_ values, the concentrations of drug required to reduce enzyme activity by 50%, were determined using GraphPad Prism 9.0 software package (GraphPad Software Inc., San Diego, CA, USA), and interpretation of IC_50_ values was performed using the WHO-AVWG (World Health Organization Expert Working Group on Influenza Antiviral Susceptibility Monitoring) criteria. The criteria for influenza A viruses were as follows: compared to the median IC_50_ value of all tested viruses by (sub)type and drug, normal inhibition was considered <10-fold, reduced inhibition was 10-to 100-fold, and highly reduced inhibition was > 100-fold (https://www.who.int/entity/wer/2012/wer8739.pdf).

### Mouse studies

Eight-week-old SPF female C57BL/6 mice (Beijing Biotechnology Co., Ltd.] were used to assess the pathogenicity of the H6 isolates. All mice were anesthetized with isoflurane. Five mice in each group were intranasally inoculated with the indicated dose of each virus in 50 μL phosphate-buffered saline (PBS). The mock group was inoculated intranasally with 50 μL of PBS. Body weights and the survival of mice were monitored daily, and mice with a 25% weight loss were euthanized for ethical reasons. At 14 dpi, serum samples were collected from the surviving mice. After being centrifuged and separated, the serum samples were treated with receptor-destroying enzyme (Denka Seiken) for 18 hours at 37°C and heat-inactivated at 56°C for 30 minutes. Then the HA inhibiting (HI) antibody titres of the samples were tested with 0.5% (vol/vol) turkey erythrocytes. Serum samples with HI titres of > 10 were classified as antibody positive. The Karber method was performed to calculate the 50% mouse infectious dose (MID_50_).

To determine the tissue tropism of the H6 viruses, mouse tissues, including the lungs, nasal turbinate, tracheal tissue, intestine tract, eyeballs and brain, were collected, and the viral titres were measured with a TCID_50_ assay in MDCK cells.

To further compare the replication abilities of the H6 viruses in infected mice, nine mice per group were inoculated intranasally with 10^5^ EID_50_ (50 µL) or 10^7^ EID_50_ (50 µL) of the indicated viruses. Three mice in each group were euthanized at 1, 3 and 5 dpi. Nasal turbinate, tracheal tissue, and lungs were collected to detect the virus titres using a TCID_50_ assay.

### Statistical analysis

The data are displayed as means ± SD. Statistical significance was determined using Student’s t-test and the GraphPad Prism 9.0 software package (GraphPad Software Inc.). A P-value < 0.05 indicated statistical significance.

## Results

### Epidemiology of H6 AIVs in China during 2000-2021

**Figure 1.**
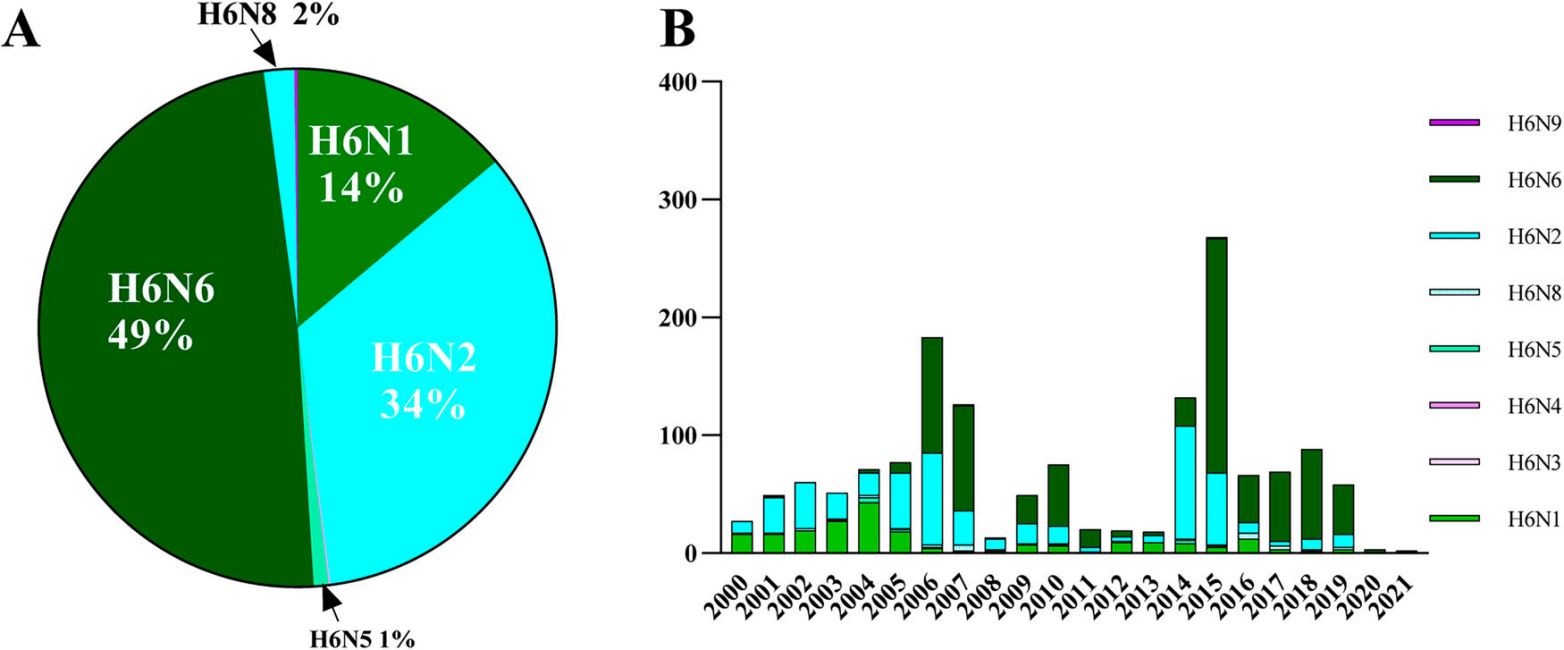
Number of H6 AIVs in China during 2000-2021. (A) General profile of H6 AIVs isolated in China from 2000 to 2021. (B) Time-dependent changes in the total number of H6 AIVs in China from 2000 to 2021.

Among all the H6 viruses, H6N6 strains accounted for the largest proportion (49%), followed by H6N2 (34%) and H6N1(14%). By contrast, the ratios of other H6 subtype viruses, including H6N8, H6N5, H6N3, H6N4 and H6N9, were low (Figure 1A). Before 2005, H6N1 and H6N2 were the major subtypes of H6 viruses. However, a rising trend has been observed in H6N6 subtype numbers since 2006. By comparison, the numbers of H6N5 and H6N8 have remained steady from 2000 to 2021 (Figure 1B). This result indicates that H6N6 was the most abundant of all H6 subtypes in the past two decades in China.

**Figure 2.**
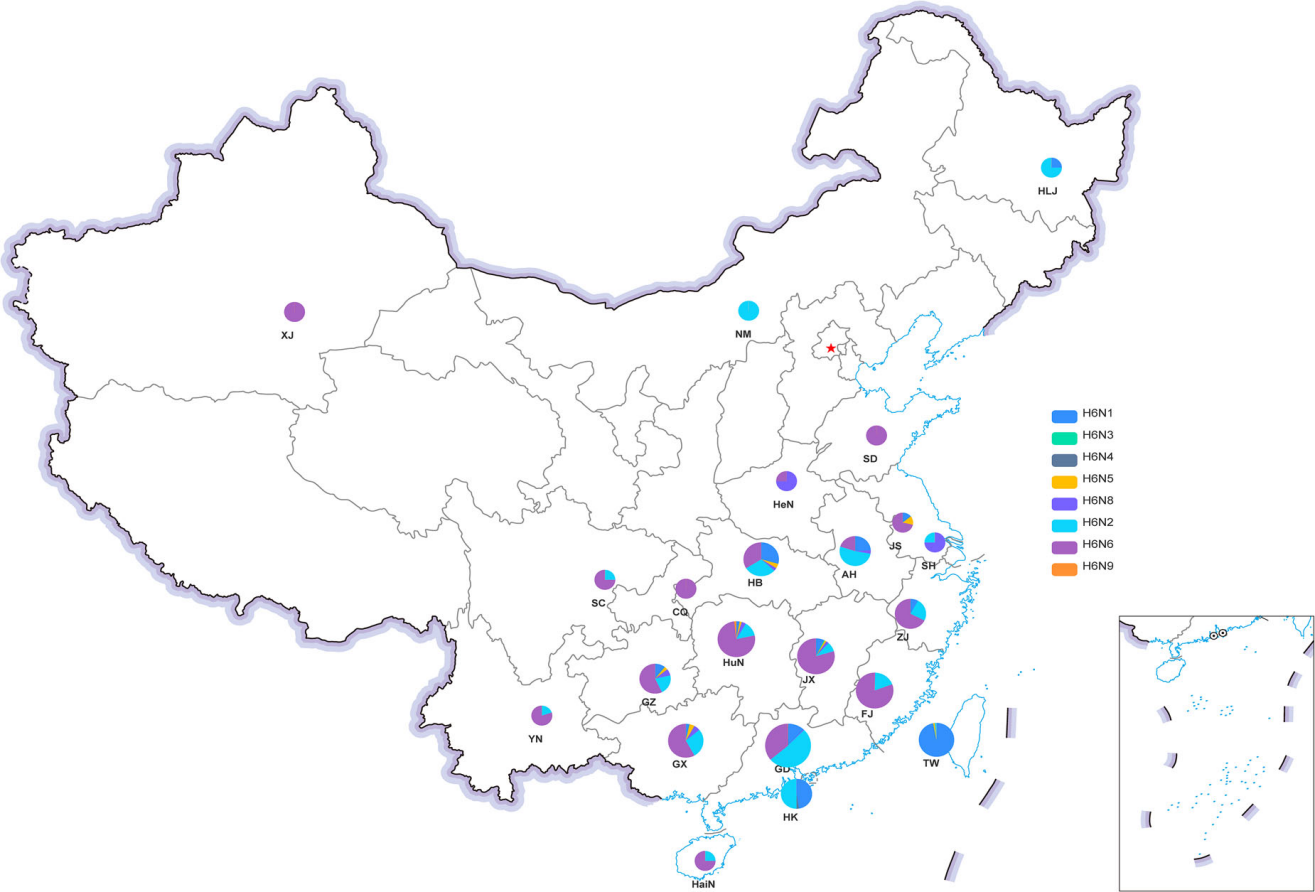
Geographical distribution of H6 AIVs in China from 2000 to 2021. Each H6 subtype is shown as a specific colour. An increase in the size of the circles indicates an increased in the number of H6 AIV incidences. XJ, Xinjiang Uygur Autonomous Region; NM, Inner Mongolia; HLJ, Heilongjiang; HeN, Henan; SD, Shandong; JS, Jiangsu; SH, Shanghai; ZJ, Zhejiang; HB, Hubei; HuN, Hunan; JX, Jiangxi; AH, Anhui; FJ, Fujian; GD, Guangdong; GX, Guangxi; HaiN, Hainan; SC, Sichuan; YN, Yunnan; GZ, Guizhou; CQ, Chongqing; HK, Hong Kong; TW, Taiwan.

Geographically, an increase in the number of H6 AIVs was observed from northern to southern China (Figure 2). We classified all the provinces that had reported H6 isolates from 2000 to 2021 into seven regions based on geographic proximity: North (Xinjiang Uygur Autonomous Region, Inner Mongolia and Heilongjiang), East-Central (Henan and Shandong), Yangtze River Delta (Jiangsu, Shanghai, and Zhejiang), Southwest (Sichuan, Yunnan, Chongqing and Guizhou), South-Central (Hubei, Hunan, Jiangxi, Anhui, and Fujian), South (Guangdong, Guangxi and Hainan), Hong Kong and Taiwan regions. The percentages of H6 isolates in these regions compared to the total number of H6 samples in all regions were 0.41%, 0.34%, 3.30%, 2.96%, 35.21%, 51.79%, and 5.98%, respectively. As shown in Figure 2, H6N6 was the major AIV subtype in the Southwest, East-Central, Yangtze River Delta, except for Anhui and Shanghai, in which H6N2 and H6N8 were predominant respectively. In contrast, both H6N6 and H6N2 were dominant in Southern China, with a relatively small proportion of H6N1. Whereas in Hong Kong and Taiwan, H6N2 and H6N1 were prominent, and there was a smaller proportion of the other subtypes (Figure 2). These results indicate that the predominant subtypes of H6Nx viruses vary among the different regions of China.

To clarify the host distributions of H6 subtype viruses, we analysed the proportion of H6 in each avian species. As shown in Figure S1A, the proportion of H6 AIVs was highest in ducks (53%), followed by environmental samples and wild fowl (14% in each), chickens (11%), geese (8%), and swine and humans (both < 1%). In terms of each H6 subtype, H6N6 exhibited the highest host diversity, being distributed in all species except for humans, followed by H6N2, H6N8 and H6N1. Chickens played a major role as H6N1 hosts, instead of ducks. Although only three H6N9 isolates were detected in China during this period, this subtype was still isolated from two host species. In addition, there was one report of H6N3 and one of H6N4 in samples from duck and wild fowls (Figure S1B). These results indicate that ducks were the most important reservoir for H6 viruses, and H6N6 viruses had the most diverse host species among all the H6Nx viruses.

### Genetic analysis of H6 AIVs in China

**Figure 3.**
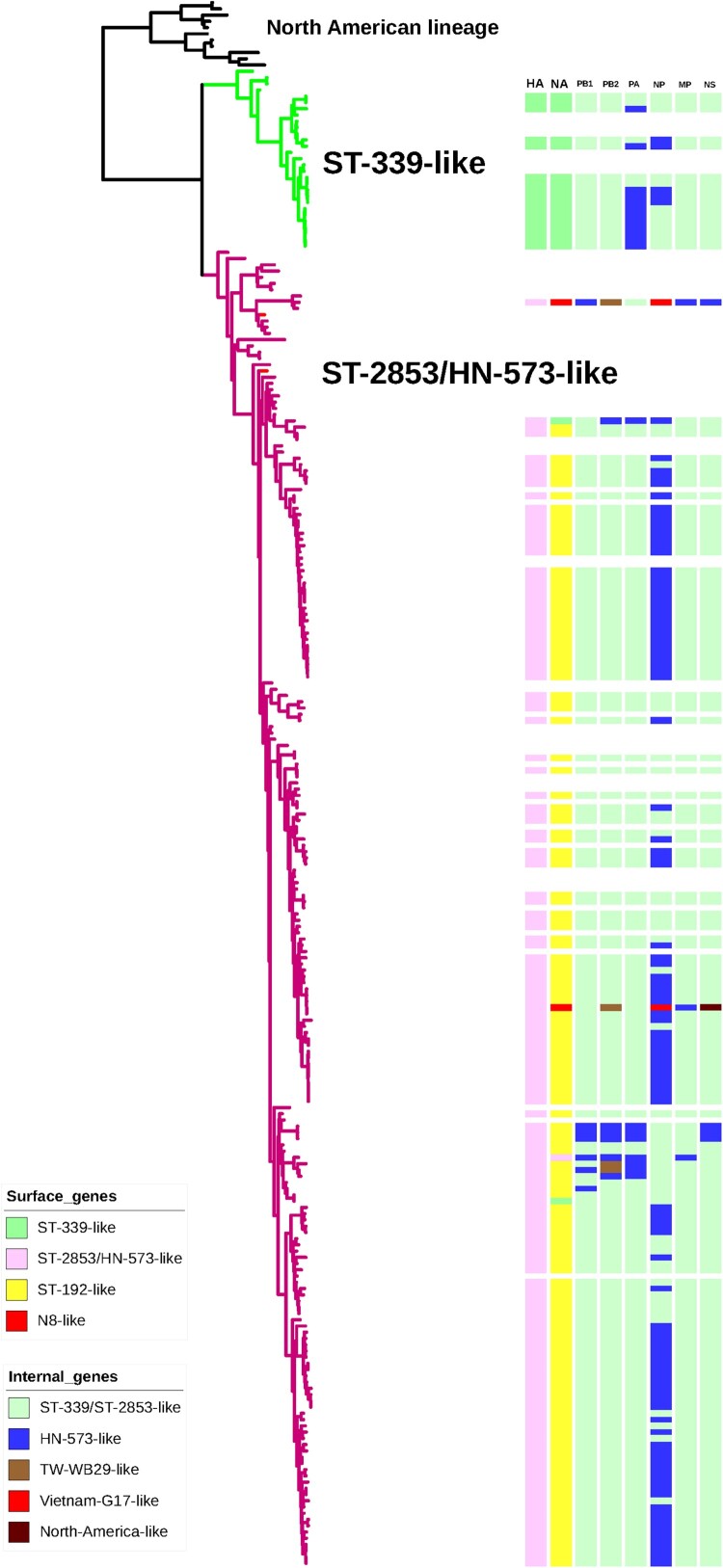
Phylogenetic tree of HA genes of H6 viruses. For the 171 H6 viruses sequenced in this study, all eight gene segments are listed at the top of the graph, and the clade origins of each gene segment are indicated by different coloured bars. White bars represent gene segments of reference strains downloaded from GISAID or GenBank. Surface genes (HA and NA) were generally classified into four separate clusters (ST-339-like, ST-2853/HN-573-like, ST-192-like and N8-like), while internal genes (PB1, PB2, PA, NP, M, NS) were included in five separate clusters.

To determine the genetic evolution of H6 viruses in China, phylogenetic analysis of all eight gene segments were conducted. As shown in Figure 3 and Supporting Information Figure S2, HA genes of all isolates fell into two separate clusters, termed ST-339-like, ST-2853/HN-573-like. The majority (90.06%, n = 154) of the H6 isolates in this study clustered into the ST-2853/HN-573-like lineage, including all 150 H6N6, 2 H6N2 and 2 H6N8 viruses. The analysis grouped 9.94% (n = 17) of H6 viruses into the ST-339-like lineage, which mostly included the H6N2 viruses.

Phylogenetic analysis of the seven genes of H6 viruses, including the NA, basic polymerase 2 (PB2), basic polymerase 1 (PB1), polymerase (PA), nucleoprotein (NP), matrix (M), and non-structural protein (NS) genes, demonstrated distinct diversity (Figure 3 and Supporting Information Figure S2). NA genes of all H6N6 strains in China from 2009 to 2021 formed a monophyletic group represented by A/wild duck/Shantou/192/2004 (H6N6) (Figure 3 and Supporting Information Figure S2). Origins of the PB1, PA, NP, M and NS genes of H6N6 viruses included ST-339/ST-2853-like and HN-573-like and several other lineages. The PB2 genes of four viruses (FJ46606, GD08951, SC05609 and SC32285) were derived from a new lineage represented by A/duck/Taiwan/WB29/99 (H6N1) (Figure 3 and Supporting Information Figure S2). These results indicated that dynamic reassortments occurred between H6N6 viruses and co-circulating AIVs in China.

### Genotypic diversity of H6Nx viruses

**Figure 4.**
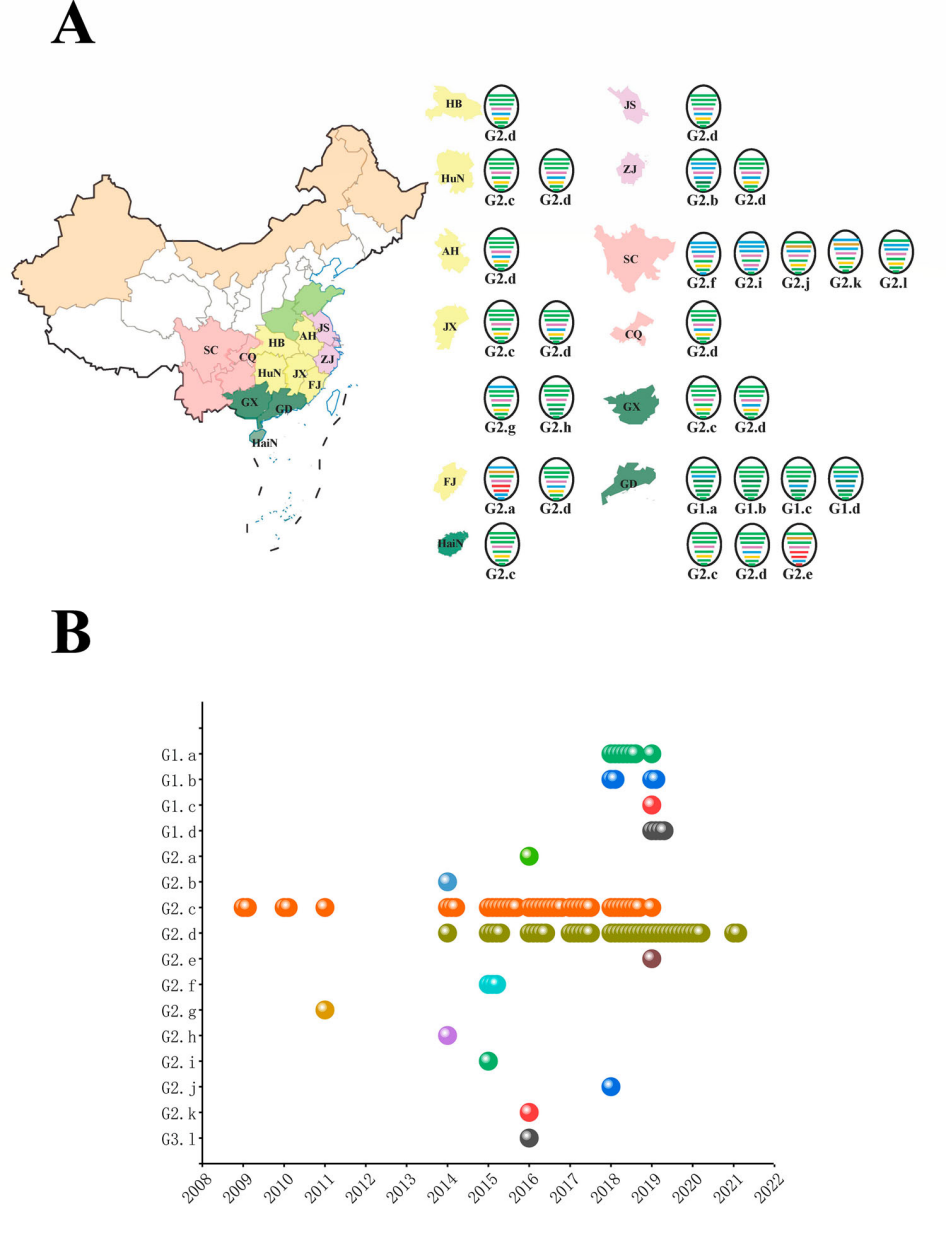
Development of H6Nx genotypes during 2009–2021. (A) Geographical distribution of H6Nx AIV genotypes in each province. The eight gene segments are shown as horizontal bars, starting from top to bottom of the virion, PB2, PB1, PA, HA, NP, NA, M and NS. Different colours represent different virus lineages. (B) Sixteen distinct genotypes are listed on the left. Coloured circles represent different genotypes and the isolation time.

According to the lineage classification of the phylogenetic trees, we designated all 171 H6 viruses collected into 16 genotypes. The viruses were generally divided into G1 and G2, which corresponded to the ST-339-like, ST-2853/HN-573-like HA lineages, respectively. Sub-genotypes G1.a–G1.d and G2.a–G2.l were further defined based on the lineages of the six internal gene segments. Moreover, one H6N8 strain formed G2.a, another H6N8 virus was of genotype G2.e, which contained the NS gene, and clustered into the North America group. Geographically, Guangdong Province had the highest number of genotypes, with up to seven genotypes (Figure 4A). In addition, Sichuan and Jiangxi ranked second and third, respectively, of regions in which the H6N6 subtype was predominantly detected (Figure 4A).

As shown in Figure 4B, G2.c was extensively prevalent during 2009–2018. In addition, G2.d was co-circulating with G2.c from 2014 to 2018 (Figure 4B). Since 2019, G2.d, accounting for 59.06% of H6 viruses, have gradually displaced G2.c genotypes to become the predominant subtype in China (Figure 4B).

### Molecular characterization

Based on the sequence analysis, we found that the vast majority of isolated H6 subtype HAs contained Q226 (168/171), G228 (170/171), E190 (171/171) and G225(171/171)(H3 numbering) at the receptor-binding sites, suggesting that these viruses have maintained a high affinity for the avian-type receptor (α2-3-SA) [20]. However, three variants, GD29711, GD30383 and ZJ21576, possessed L226, and one variant, GD29646, possessed S228, indicating their high affinity for the human-type receptor (α2-6-SA) (Table S1). In addition, all 171 H6 AIVs had the same single basic amino acid in the cleavage site (PQIETR↓GL) between HA1 and HA2, which represents a low-pathogenic avian influenza virus feature.

In the NA stalk region, two H6N6 isolates (JX24/09 and HN01/09) possessed a 27-nucleotide deletion, resulting in the loss of nine amino acids (Table S1) that might be associated with increased virulence in mammals [21]. Moreover, 21.9% (33/151) of the H6N6 isolates had an 11-amino acid deletion in the stalk region (positions 59–69); five of these viruses (yellow-labelled) were tested for their pathogenicity in mice, and they did not cause weight loss or death (Table S1).

Amino acid changes in PB2, including E627 K and D701N, contribute to the increased virulence and transmission of influenza viruses in mammals [22]. In our study, none of the 171 H6 viruses had these mutations (Table S1). There were no amino acid mutations in PB1 or PA proteins related to increased virulence in mammals (Table S1).

Substitutions which were reported to be associated with drug resistance to NAIs [23] were not observed in all H6 viruses. In addition, six H6 AIVs were randomly selected for NA inhibition assay, and all were sensitive to oseltamivir (Figure S6, Table S2).

### Pathogenic characterization of H6N6 viruses

Based on the genotype classification, a total of 29 representative viruses were selected for the pathogenicity characterization study. Each virus was inoculated into 8-week-old female C57B/L6 mice intranasally at 10^6^ EID_50_ in 50 μl PBS. Mice were inoculated with PBS as the control. The survival condition and bodyweight loss of these mice were monitored for 14 days. None of the mice died, and the body weight loss was <20% for those inoculated with all selected viruses at 10^6^ EID_50_ (Figure S3)_._ However, the bodyweight of mice infected with JX24/09 and HN/28868 decreased significantly (Figure S3), suggesting that these H6N6 AIVs may present different pathogenic phenotypes in mice.

**Figure 5.**
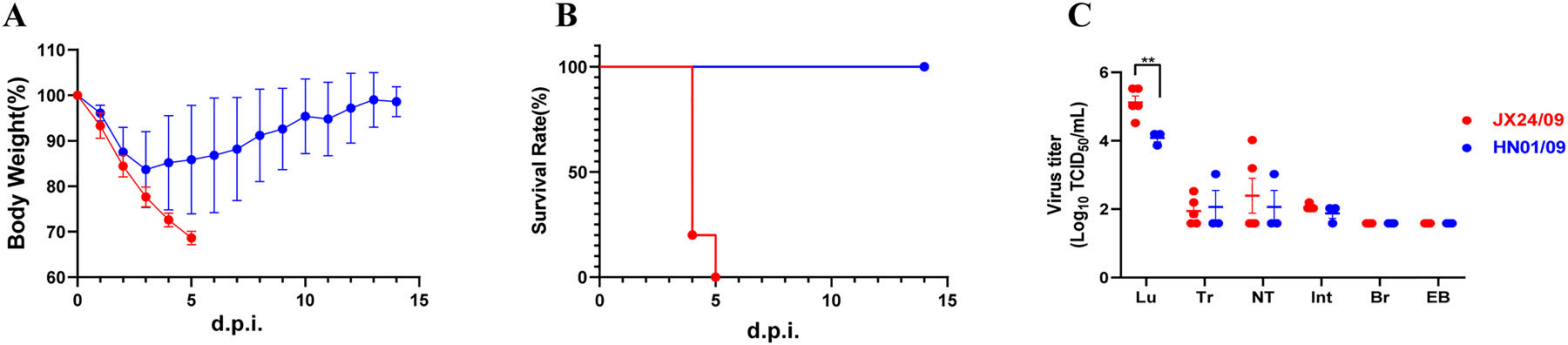
H6N6 AIV characterization in mice Bodyweight loss (A) and survival rate (B) of mice infected with various H6N6 strains. Groups of mice (n = 5) were intranasally infected with 10^7^ EID_50_ of each virus. Bodyweight loss and survival of mice were monitored until 14 dpi. Statistical significance of the survival curve was determined using a Log-rank (Mantel–Cox) test. (C) Viral replication efficiency in the different tissues of mice was analysed by inoculating mice (n = 5 per group) intranasally with 10^7^ EID_50_ of each virus.

We further infected mice intranasally with JX24/09 and HN01/09 as controls at the dose of 10^7^ EID_50_. The survival and bodyweight loss of the animals were monitored until 14 dpi. As shown in Figure 5A-B, JX24/09 caused illness and obvious bodyweight loss of mice from 1 dpi, and led to fatality rates of 100%. In contrast, all mice infected with HN01/09 survived and only had a small loss of bodyweight (Figure 5A-B).

Tissues, including lung, tracheal tissue, nasal turbinate, intestine tract, brain, and eyeballs, were collected from infected mice at 5 dpi. Viruses were detected only in the respiratory tissues, including lung, tracheal and nasal turbinate tissues. These results indicate that H6N6 viruses preferentially infected the respiratory system, rather than extra-pulmonary tissues.

Although viral titres in the tracheal tissue and nasal turbinate tissues infected with JX24/09 virus were similar to those of HN01/09, the viral titre in lung tissue infected with JX24/09 was significantly higher than that of HN01/09 (p < 0. 01) (Figure 5C). These data suggest that JX24/09 replicated more efficiently than HN01/09 in the lung tissue of mice but similarly to HN01/09 in the upper airway.

### Virulence, replication and infectivity of JX24/09, HN01/09 and GD30383 in mice

**Figure 6.**
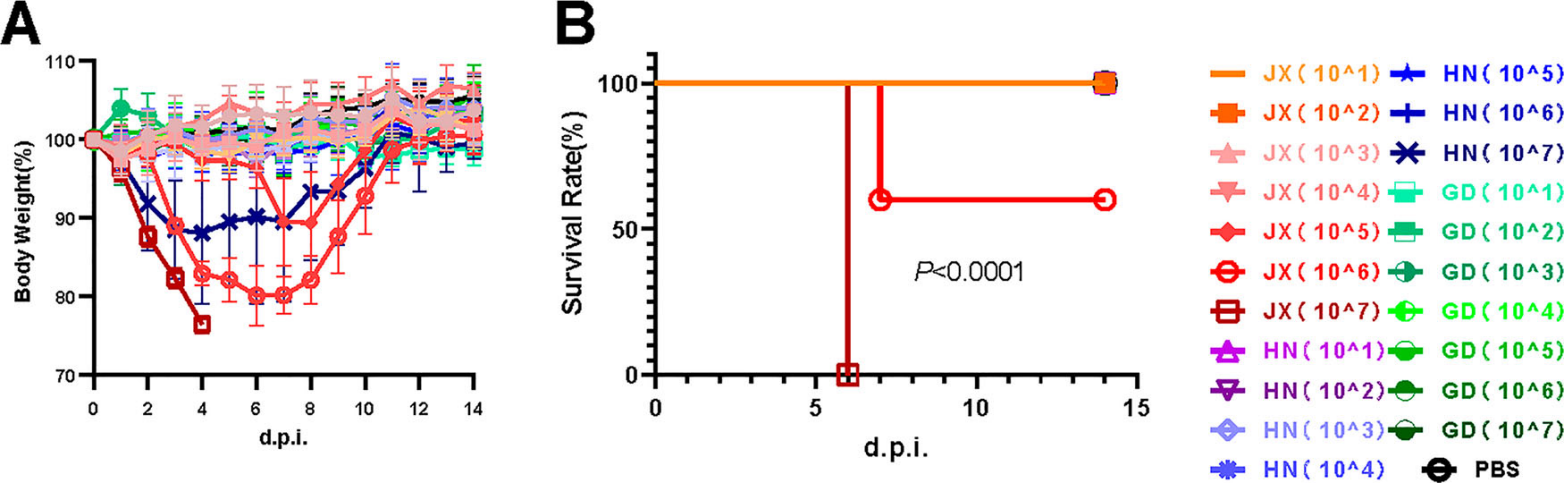
Virulence and replication of JX24/09 and HN01/09 in mice. Five mice per group were inoculated intranasally with PBS (control) or with 10^1^–10^7^ EID_50_ of JX24/09 or HN01/09. Bodyweight changes (A) and survival of mice (B) were monitored daily. Statistical significance of the survival curve was determined using a Log-rank (Mantel–Cox) test.

To investigate the pathogenicity of the two representative H6N6 viruses (JX24/09 and HN01/09) in detail, their virulence, replication and infectivity were evaluated *in vivo* and *in vitro*. We also included a control H6N6 virus (GD30383) that does not have NA stalk deletions to study its virulence, replication, and infectivity compared with JX24/09 and HN01/09. As shown in Figure 6, when inoculated with 10^1^–10^5^ EID_50_ of JX24/09 or HN01/09 virus, no mice died, and the bodyweight loss was < 20% (Figure 6A). However, when infected with 10^6^–10^7^ EID_50_ of JX24/09, bodyweight loss was dramatically greater. Additionally, the 50% mouse lethal dose (MLD_50_) value of JX24/09 (10^6.1^EID_50_) was 25 times lower than that of HN01/09 (≥10^7.5^EID_50_) and GD30383 (≥10^7.5^ EID_50_), which was expected (Figure 6B).

According to the results of the HI antibody test, the MID_50_ value of JX24/09 was 10^3.3^EID_50_, which was almost 63-fold lower than that of HN01/09 (10^5.1^EID_50_) and 16-fold lower than that of GD30383(10^4.5^EID_50_) (Table 1). These data demonstrated the higher virulence of the JX24/09 virus compared with HN01/09 and GD30383 in mice.

**Table 1.** Hemagglutinin inhibition antibody test of the C57BL/6 mice inoculated with JX/24/09 and HN/01/09.

Viruses	Inoculation dose (log10 EID_50_)	HI titers					MID_50_ (log10 EID_50_)
		#1	#2	#3	#4	#5	
JX 24 09	7	40	ND	ND	ND	ND	3.3
	6	20	20	80	20	20	
	5	10	160	20	20	20	
	4	10	10	10	<10	<10	
	3	<10	<10	10	10	20	
	2	<10	<10	<10	<10	<10	
	1	<10	<10	<10	<10	<10	
HN 01 09	7	10	40	20	20	80	5.1
	6	10	<10	40	10	10	
	5	<10	<10	<10	20	20	
	4	<10	<10	10	<10	<10	
	3	<10	<10	<10	<10	<10	
	2	<10	<10	<10	<10	<10	
	1	<10	<10	<10	<10	<10	
GD30383	7	80	80	40	80	80	4.5
	6	160	160	160	80	<10	
	5	80	80	20	40	20	
	4	<10	<10	20	<10	<10	
	3	<10	<10	<10	<10	<10	
	2	<10	<10	<10	<10	<10	
	1	<10	<10	<10	<10	<10	
PBS		<10	<10	<10	<10	<10	

Notes: (1) Serums were collected from each survival mouse (#1 to #5, respectively) at 14 dpi, and were tested for HI titers against JX/24/09 virus. MID_50_ was determined using the Karber Method.

(2) ND: Not determined due to death of the animal (treated as positive for determination of the MID_50_). HI titer < 10 was regarded as negative for seroconversion.

When inoculated with 10^5^ EID_50_ of virus, the titres of the HN01/09 and GD30383 viruses were significantly lower than those of JX24/09 virus in the lung tissues of mice at 1, 3 and 5 dpi and tracheal tissues of mice at 5 dpi (p < 0.01) (Figure S4A). Although the HN01/09 virus exhibited a trachea viral titre slightly higher than that of JX24/09 at 5 dpi at the dose of 10^7^ EID_50_, the nasal turbinate viral titre of JX24/09 was higher than that of HN01/09 (p < 0.01) (Figure S4B). Furthermore, at 1 dpi, the viral titres of JX24/09 were higher than those of HN01/09 in lung and nasal turbinate (p < 0.01) (Figure S4B). These data suggested that JX24/09 replicated more efficiently in the upper airway and lungs of mice than HN01/09 and GD30383.

The growth-kinetic properties of the three viruses in MDCK cells were also compared. Data showed (Figure S5) that the two selected viruses had different replication abilities at 37°C. JX24/09 replicated efficiently and resulted in a higher viral titre than HN01/09, which had the lowest titre at most timepoints (6-36 hpi) (P < 0.05). In contrast, GD30383 replicated poorly at all timepoints. In conclusion, JX24/09 replicated rapidly in MDCK cells and maintained higher viral titres than that of HN01/09 and GD30383, which was consistent with the tissue tropism results.

## Discussion

H6 AIVs have been sporadically reported to infect different species of wild birds and domestic poultry during their cocirculation around the world. They have caused several pandemic threats, including H7N9, H9N2 and H5N1 virus outbreaks, for many years [22,24–27], promoting interactions or gene exchanges and representing a clear threat to public health [5,10]. Thus, systemic analyses and evaluations of the epidemiology, evolution, and biological characteristics of H6 AIVs in China are imperative.

In this study, we conducted a comprehensive prevalence analysis of H6 AIVs for the first time, using 1524 sequences obtained from 2000 to 2021. H6 subtype AIVs were widely detected throughout 21 provincial regions in China (Figure 2), and the increasing trend in the number of H6 AIVs from Northern to Southern China is similar to that of other AIVs, such as the H9N2 virus [28]. During previous investigations, short-stalk NA was proven to be a strong determinant of the adaptation of aquatic bird influenza viruses to poultry and has appeared more and more frequently [29]. Somewhat simultaneously, H6N6 viruses began to replace H6N2 viruses, eventually becoming the most predominant subtype in China (Figure 1B). Based on this, we suspected that the most likely driving force behind this phenomenon was the appearance of an increasing number of H6N6 viruses with shortened NA stalks, which could be better able to adapt to poultry species in China.

Based on the results of a study that showed ducks were the main hosts of H6 viruses, and the previous conclusion that wild waterfowl were the natural reservoirs that contributed to the geographical spread of AIVs via long-distance migration [19], we speculated that the high number of H6 AIVs may partially be due to the high density setting, but free-range manner, of live poultry farming. This setting provides an environment that facilitates close contact between migratory birds and domestic poultry, in which they share food, water, and even habitats, creating more opportunities for occasional viral transmission between wildfowl and domestic poultry. Often, ducks do not show any symptoms of H6 AIV infection, which is more conducive to viruses being transmitted from ducks to other hosts, increasing the possibility of animal and human infections, and providing a breeding ground for viral reassortment. Therefore, to disrupt the chain of transmission from wild birds, improvements in biosafety measures and poultry feeding patterns in poultry farms seem necessary. However, although we have conducted in-depth research into H6 AIVs, we must admit that some sample bias, in terms of the limited number of sequences and the range of geographical and host coverage was unavoidable. This is likely to have resulted in inadequate profiles of the emergence and evolution of H6 viruses. For example, for data from public databases, different researchers may sample according to their own research purposes, which led to a sampling bias in the uploaded data, not representing the overall profile of H6 AIVs. Besides, the number of H6 strains has decreased sharply since 2020, which may be affected by the outbreak of SARS-CoV-2, and the sampling and transportation of strains have been restricted.

In our study, the genotypic diversity of H6 viruses and 16 genotypes was revealed in the genome analysis, providing an indication of the intricate reassortment that occurs among these viruses during their active circulation with other viruses. The same characteristics were seen for the highly pathogenic avian influenza (HPAI) H5N1 virus and the H7N9 virus. For instance, during the 1997 outbreak in Hong Kong, H5N1 influenza viruses identified in this region were considered to be complicated reassortants that acquired the ability to infect and kill mammals gradually [26]. Previously, reassortment has contributed to the emergence and spread of pandemic influenza viruses in human populations [30]. However, an H3N8 AIV jumped the species barrier recently and caused acute respiratory distress syndrome in a child. Consequently, it was extremely likely to be a novel reassortant bearing certain genes from other influenza viruses [31]. Even though human-derived gene cassettes have not yet been reported in H6 AIVs, clearly, the possibility that such reassortments will occur someday exists.

H6 AIVs had been found to replicate in mice without preadaptation, but with less weight loss and an absence of death [32]. In this study, several of the H6N6 viruses caused severe clinical symptoms in mammals or eventual death. It has been demonstrated that a deletion in the NA stalk region increased the virulence of waterfowl-derived influenza viruses in poultry [29]. Interestingly, the two H6N6 isolates (JX24/09 and HN01/09), despite having totally different pathogenicities in mice (Figure 5A-B), shared the same nine amino acids deficit in this region and even possessed completely identical NA sequences. Hence, the real determinant of virulence and the molecular mechanism underlying the difference in the biological characteristics of H6 viruses deserve thorough explorations. Moreover, molecular analysis suggested that the Q226L and G228S mutations existed in the HA proteins of our isolates and may have contributed to their increased affinity for the human-type receptor. Taken together, our findings suggest that H6 viruses pose an increasingly serious threat to public health.

In summary, given that H6 viruses have acquired the ability to replicate in mammalian hosts, we cannot rule out the possibility that they will eventually evolve into viruses with efficient transmissibility in mammals and even human populations via the accumulation of reassortments or mutations. Therefore, long-term surveillance of H6 AIVs in China is urgently needed.

## Supplementary Material

Supplemental Material
