# Supplementary material for: Epidemiology, evolution, and biological characteristics of H6 avian influenza viruses in China
Source: Emerg Microbes Infect. 2022 Dec 20;12(1):2151380. doi: 10.1080/22221751.2022.2151380 (PMC9788695; doi:10.1080/22221751.2022.2151380)
Supplement: Supplemental Material [file TEMI_A_2151380_SM4152.zip › Table S1.docx]

**Table S1. Molecular characteristics of H6 viruses**

| **Isolate** | **Amino acid sequence**  **at cleavage site of HA** | **Receptor-binding**  **sites in HA** | | **Amino acid deletion in NA (position)** | **PA** | **PB1** | **PB2** | | | | |
| --- | --- | --- | --- | --- | --- | --- | --- | --- | --- | --- | --- |
|  |  | **Q226L** | **G228S** |  | **R192H** | **A453S** | **R17C** | **E158G** | **A271T** | **E627K** | **D701N** |
| JX24/09 | PQIETR↓GLF | Q | G | Yes (60–68) | R | A | R | E | A | E | D |
| HN01/09 | PQIETR↓GLF | Q | G | Yes (60–68) | R | A | R | E | A | E | D |
| GD29711 | PQIETR↓GLF | L | G | NO | R | A | R | E | A | E | D |
| GD29646 | PQIETR↓GLF | Q | S | NO | R | A | R | E | A | E | D |
| GD30383 | PQIETR↓GLF | L | G | NO | R | A | R | E | A | E | D |
| ZJ21576 | PQIETR↓GLF | L | G | NO | R | A | R | E | A | E | D |
| GD09103 | PQIETR↓GLF | Q | G | Yes (59–69) | R | A | R | E | A | E | D |
| GD09104 | PQIETR↓GLF | Q | G | Yes (59–69) | R | A | R | E | A | E | D |
| GD09096 | PQIETR↓GLF | Q | G | Yes (59–69) | R | A | R | E | A | E | D |
| GD09099(yellow-labelled) | PQIETR↓GLF | Q | G | Yes (59–69) | R | A | R | E | A | E | D |
| GD09003 | PQIETR↓GLF | Q | G | Yes (59–69) | R | A | R | E | A | E | D |
| GD09002 | PQIETR↓GLF | Q | G | Yes (59–69) | R | A | R | E | A | E | D |
| FJ40677 | PQIETR↓GLF | Q | G | Yes (59–69) | R | A | R | E | A | E | D |
| JS35713(yellow-labelled) | PQIETR↓GLF | Q | G | Yes (59–69) | R | A | R | E | A | E | D |
| FJ40782 | PQIETR↓GLF | Q | G | Yes (59–69) | R | A | R | E | A | E | D |
| FJ46627 | PQIETR↓GLF | Q | G | Yes (59–69) | R | A | R | E | A | E | D |
| JS06680 | PQIETR↓GLF | Q | G | Yes (59–69) | R | A | R | E | A | E | D |
| GD34150 | PQIETR↓GLF | Q | G | Yes (59–69) | R | A | R | E | A | E | D |
| GD34110 | PQIETR↓GLF | Q | G | Yes (59–69) | R | A | R | E | A | E | D |
| GD14489 | PQIETR↓GLF | Q | G | Yes (59–69) | R | A | R | E | A | E | D |
| HN25033 | PQIETR↓GLF | Q | G | Yes (59–69) | R | A | R | E | A | E | D |
| HN24897 | PQIETR↓GLF | Q | G | Yes (59–69) | R | A | R | E | A | E | D |
| FJ39246 | PQIETR↓GLF | Q | G | Yes (59–69) | R | A | R | E | A | E | D |
| FJ24908 | PQIETR↓GLF | Q | G | Yes (59–69) | R | A | R | E | A | E | D |
| GD48556(yellow-labelled) | PQIETR↓GLF | Q | G | Yes (59–69) | R | A | R | E |  | E | D |
| JX42952 | PQIETR↓GLF | Q | G | Yes (59–69) | R | A | R | E | A | E | D |
| JX42947 | PQIETR↓GLF | Q | G | Yes (59–69) | R | A | R | E | A | E | D |
| ZJ21566 | PQIETR↓GLF | Q | G | Yes (59–69) | R | A | R | E | A | E | D |
| GD47376 | PQIETR↓GLF | Q | G | Yes (59–69) | R | A | R | E | A | E | D |
| AH38989(yellow-labelled) | PQIETR↓GLF | Q | G | Yes (59–69) | R | A | R | E | A | E | D |
| GD54427(yellow-labelled) | PQIETR↓GLF | Q | G | Yes (59–69) | R | A | R | E | A | E | D |
| FJ46690 | PQIETR↓GLF | Q | G | Yes (59–69) | R | A | R | E | A | E | D |
| HB43311 | PQIETR↓GLF | Q | G | Yes (59–69) | R | A | R | E | A | E | D |
| GD29644 | PQIETR↓GLF | Q | G | Yes (59–69) | R | A | R | E | A | E | D |
| GD29646 | PQIETR↓GLF | Q | G | Yes (59–69) | R | A | R | E | A | E | D |
| GD29648 | PQIETR↓GLF | Q | G | Yes (59–69) | R | A | R | E | A | E | D |
| GD29652 | PQIETR↓GLF | Q | G | Yes (59–69) | R | A | R | E | A | E | D |
| FJ06277 | PQIETR↓GLF | Q | G | Yes (59–69) | R | A | R | E | A | E | D |
| FJ32132 | PQIETR↓GLF | Q | G | Yes (59–69) | R | A | R | E | A | E | D |

Notes: Variant amino acids in the HA, NA, PA, PB1 and PB2 proteins are shown for sequences. Yellow-labeled strains were tested for pathogenicity in mice.
