# Supplementary material for: Epidemiology, evolution, and biological characteristics of H6 avian influenza viruses in China
Source: Emerg Microbes Infect. 2022 Dec 20;12(1):2151380. doi: 10.1080/22221751.2022.2151380 (PMC9788695; doi:10.1080/22221751.2022.2151380)
Supplement: Supplemental Material [file TEMI_A_2151380_SM4152.zip › Table_S2.docx]

Table S2. Drug susceptibility of H6 AIVs in China during 2000-2021.

| Isolate name | Oseltamivir | |
| --- | --- | --- |
|  | IC_50_ (nM)^a^ | Fold change^b^ |
| GD29711 | 1.03 | 2.48 |
| GD30383 | 0.911 | 2.08 |
| ZJ21576 | 0.4074 | 0.38 |
| GD29646 | 0.8698 | 1.94 |
| JX2409 | 0.6445 | 1.18 |
| HN0109 | 0.9264 | 2.13 |
| Resistant control | 26.74 | 1 |
| Sensitive control | 0.296 | 1 |

Notes:

a IC50, half-maximal inhibitory concentration. The IC50 denotes the concentration of a NA inhibitor that reduces the NA activity by 50% relative to NA activity without the inhibitor.

b Fold change relative to the mean IC50 of the wild-type NA protein. The IC50 of wild-type NA protein means the average IC50 values of the wild type viruses, without any mutations. Fold-change values of each NA were interpreted using criteria established by the World Health Organization Influenza Antiviral Working Group. The criteria for influenza A viruses: compared to the median IC50 value of all tested viruses by (sub)type and drug, normal inhibition (NI) with <10-fold, reduced inhibition (RI) with 10-to 100-fold, and highly reduced inhibition (HRI) with > 100-fold.
