# Supplementary figures and images for: Epidemiology, evolution, and biological characteristics of H6 avian influenza viruses in China
Source: Emerg Microbes Infect. 2022 Dec 20;12(1):2151380. doi: 10.1080/22221751.2022.2151380 (PMC9788695; doi:10.1080/22221751.2022.2151380)

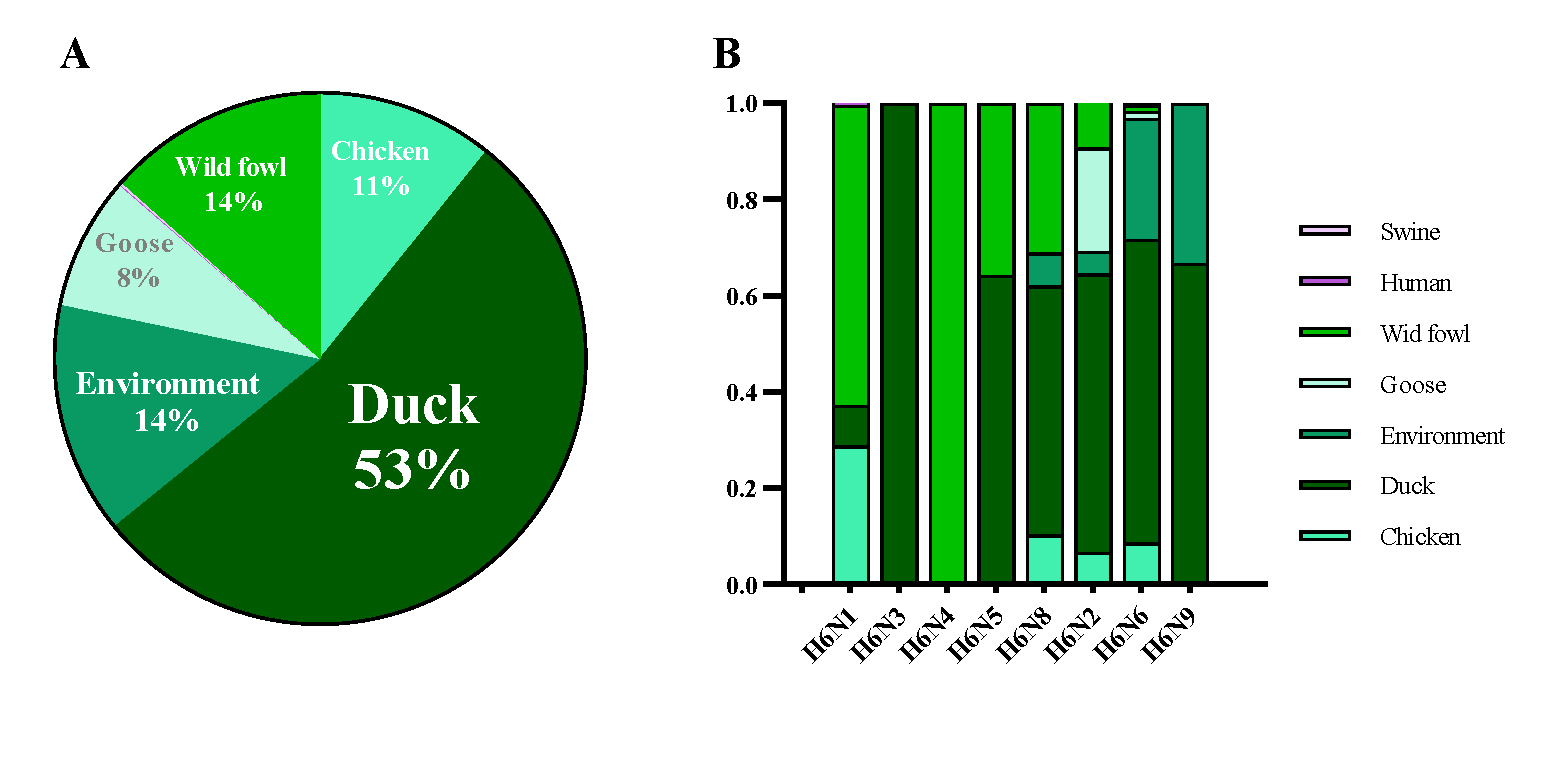

Supplement: Supplemental Material [file TEMI_A_2151380_SM4152.zip › Figure S1.tif]

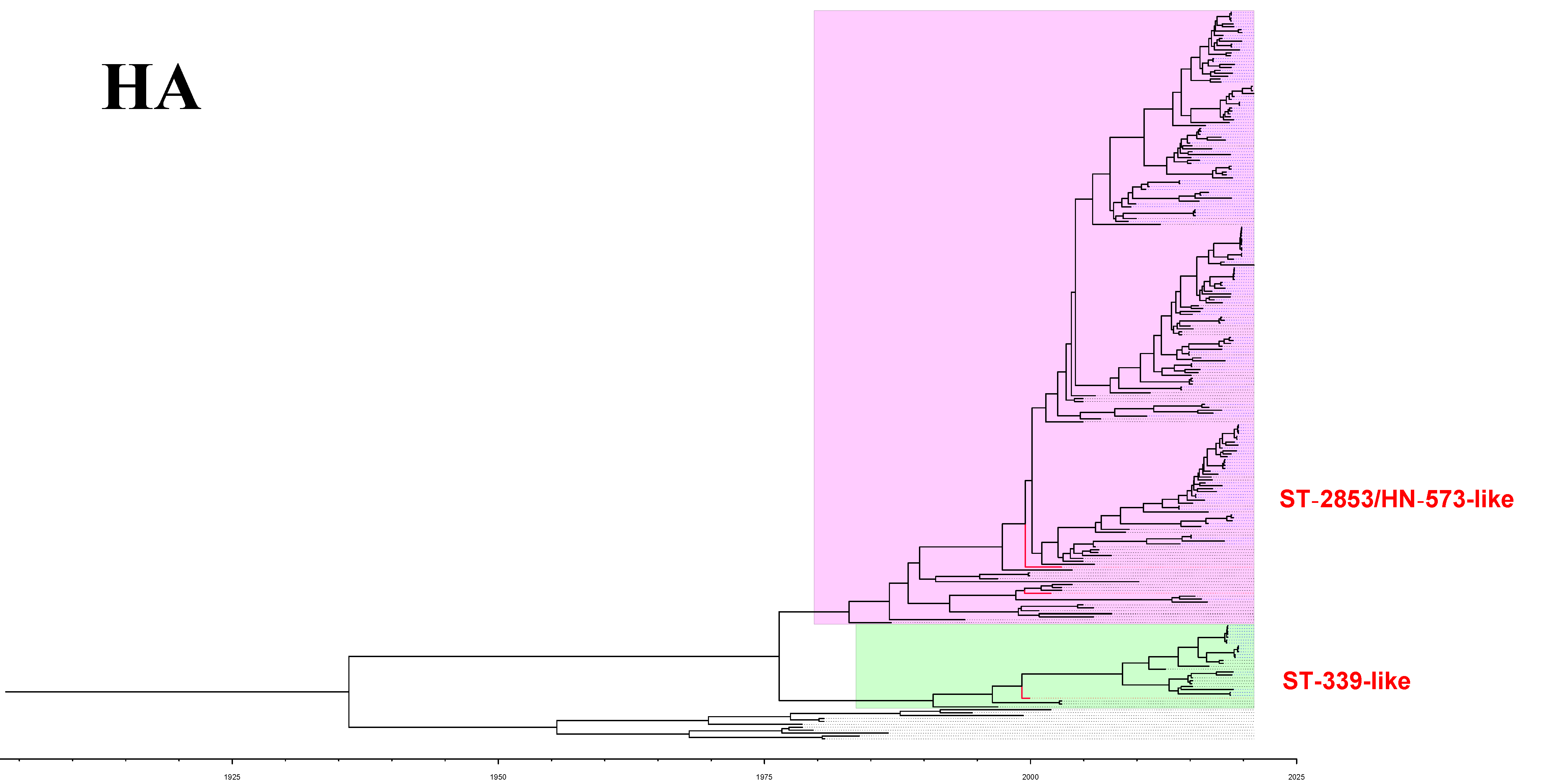

Supplement: Supplemental Material [file TEMI_A_2151380_SM4152.zip › Figure S2 _HA.tif]

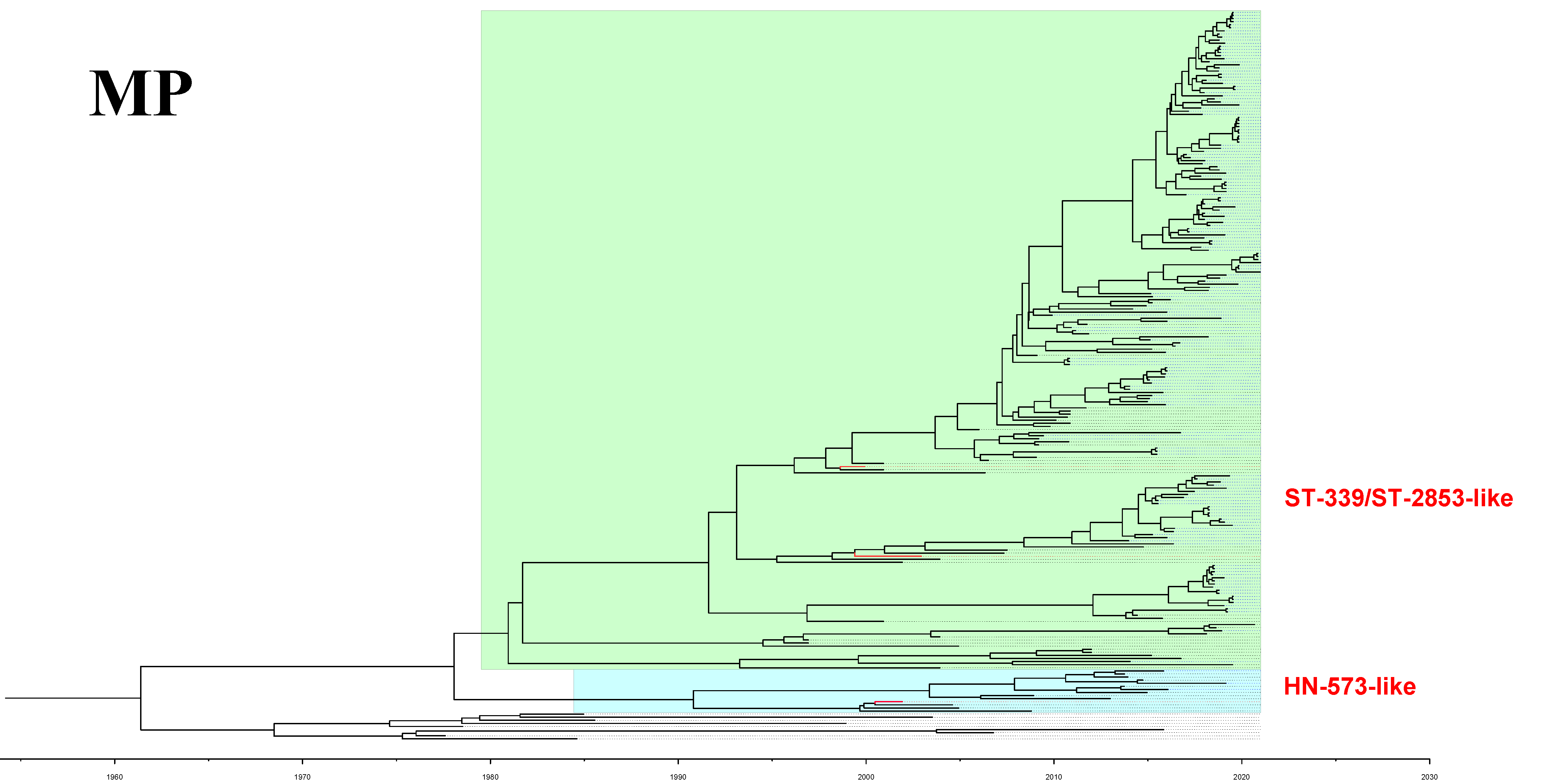

Supplement: Supplemental Material [file TEMI_A_2151380_SM4152.zip › Figure S2 _MP.tif]

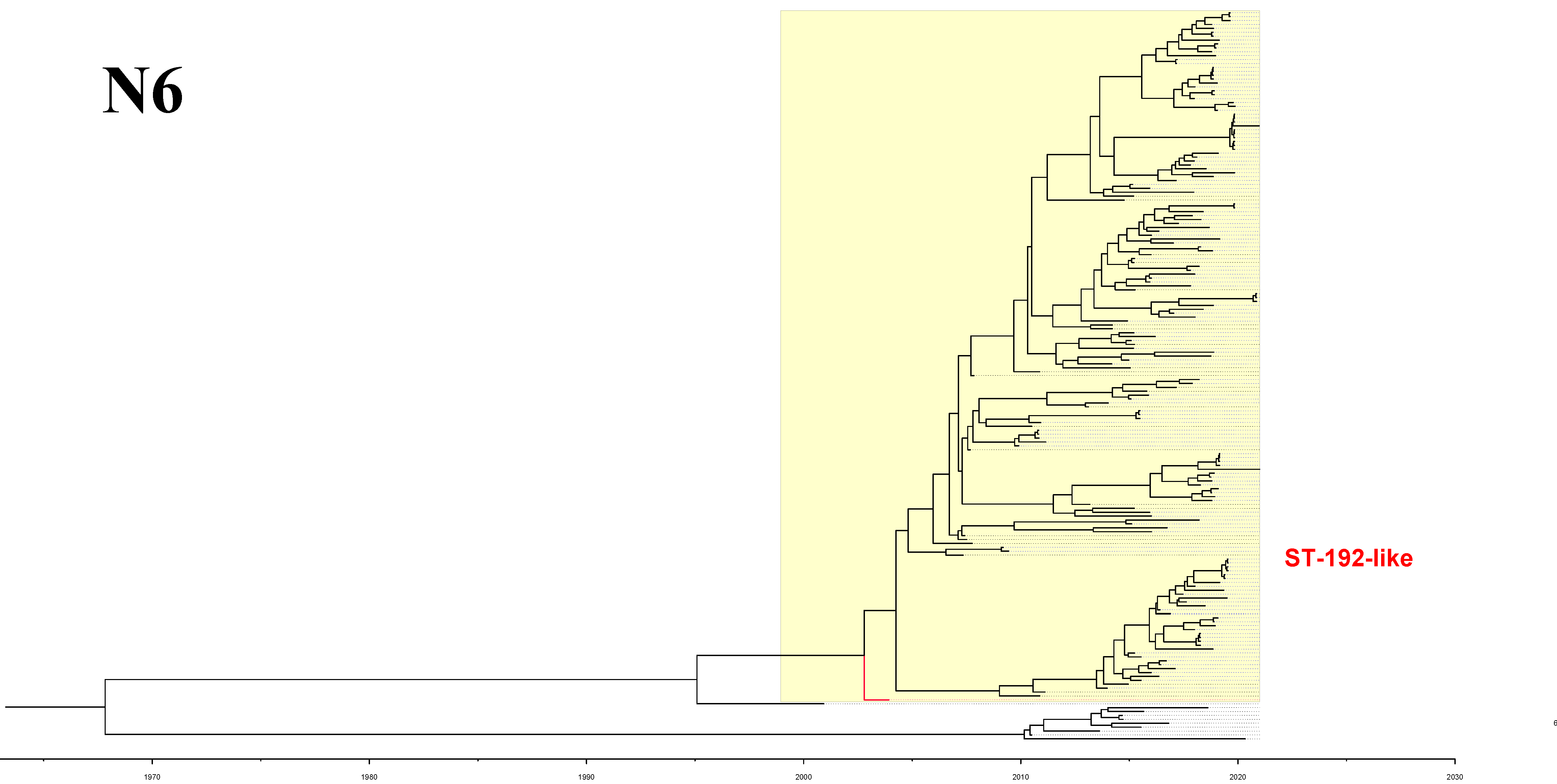

Supplement: Supplemental Material [file TEMI_A_2151380_SM4152.zip › Figure S2 _N6.tif]

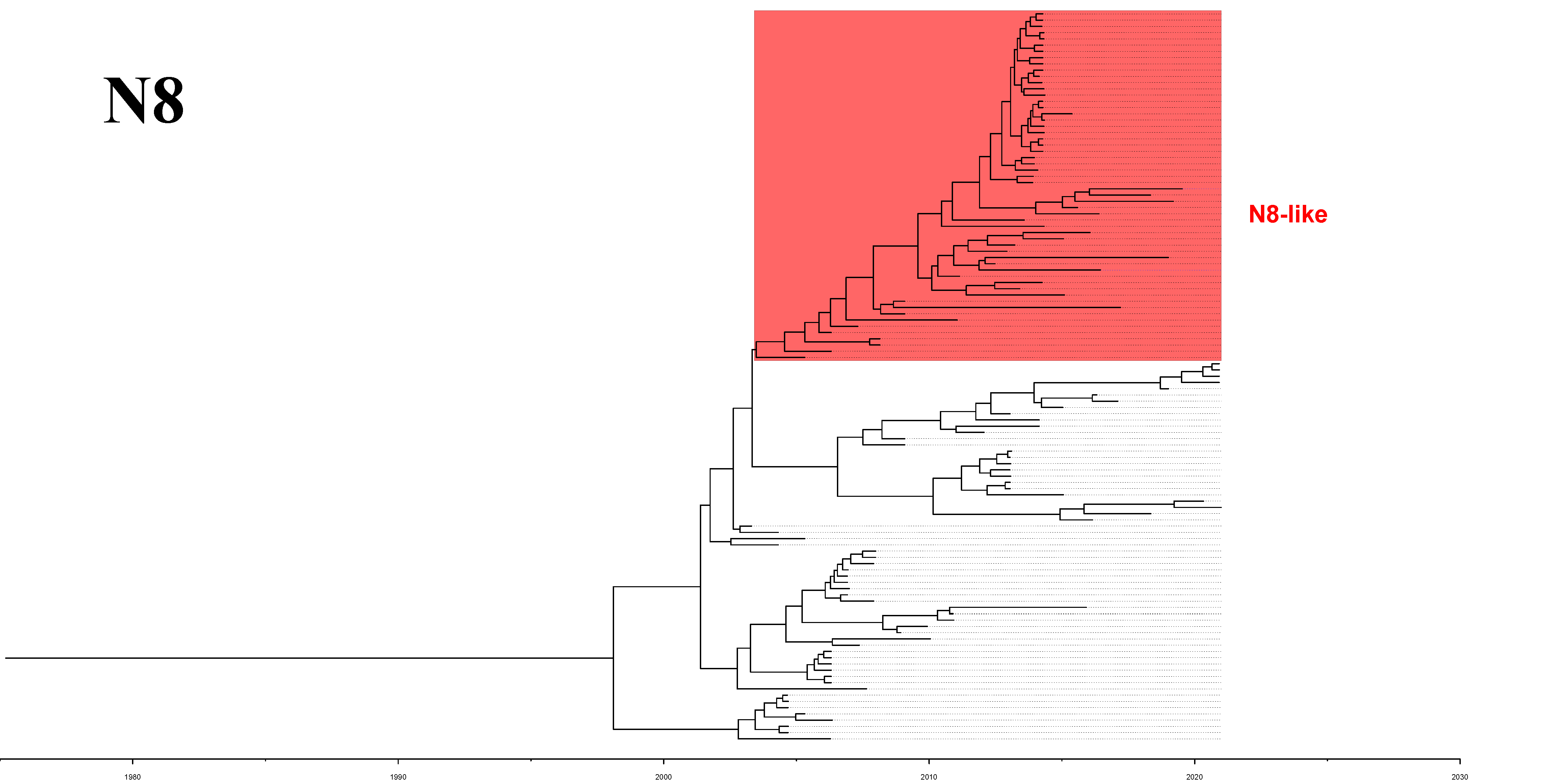

Supplement: Supplemental Material [file TEMI_A_2151380_SM4152.zip › Figure S2 _N8.tif]

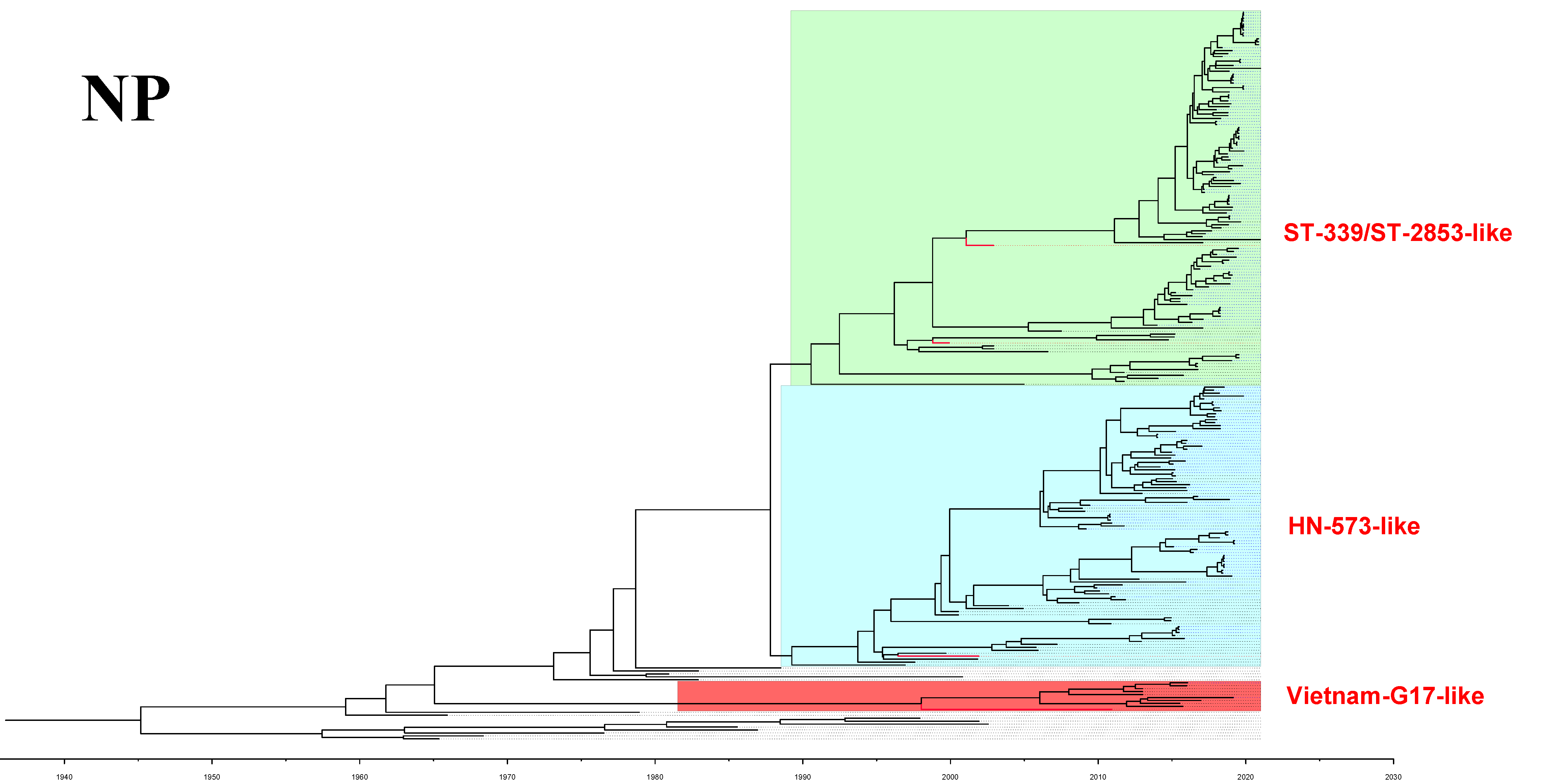

Supplement: Supplemental Material [file TEMI_A_2151380_SM4152.zip › Figure S2 _NP.tif]

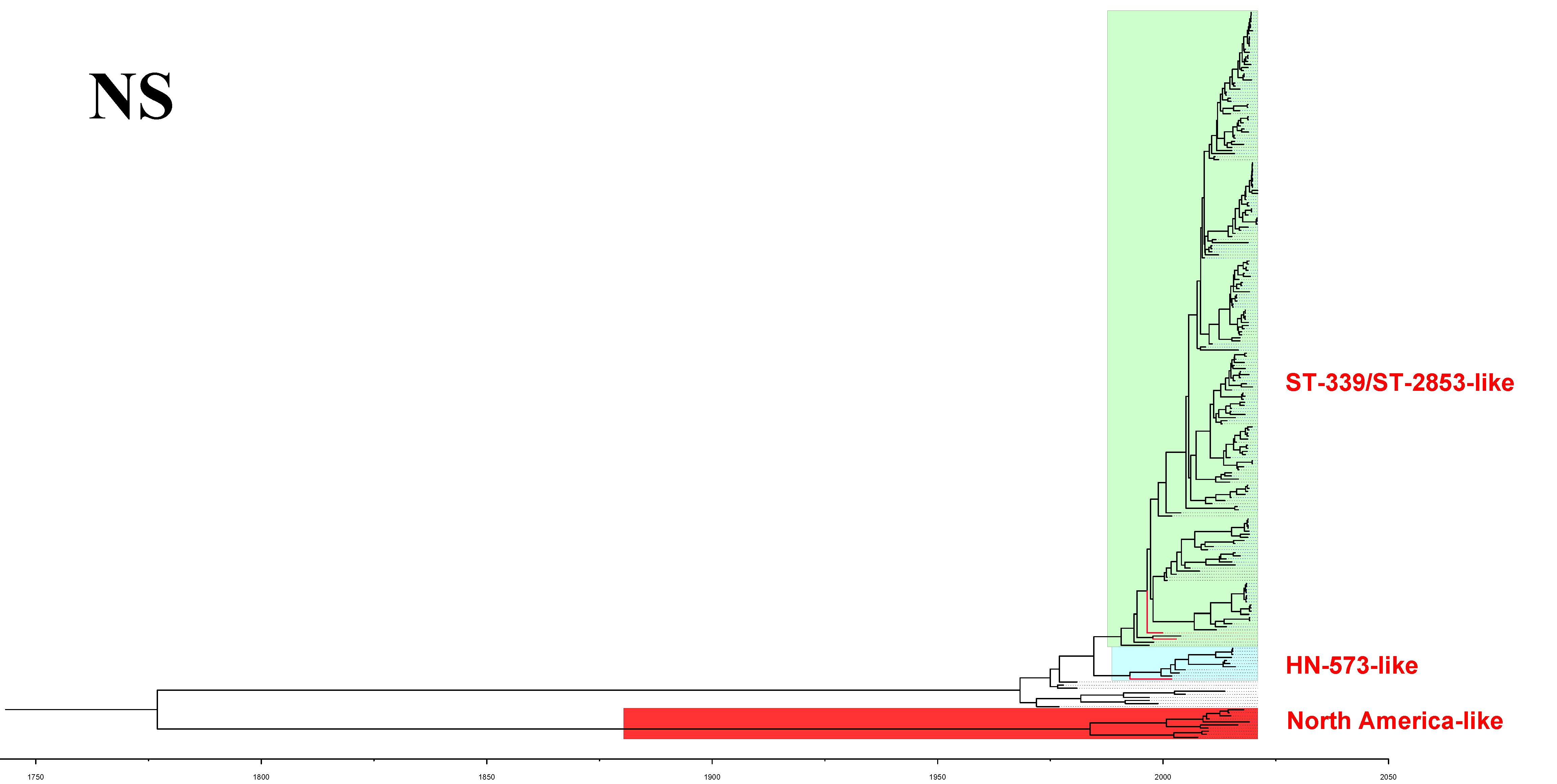

Supplement: Supplemental Material [file TEMI_A_2151380_SM4152.zip › Figure S2 _NS.tif]

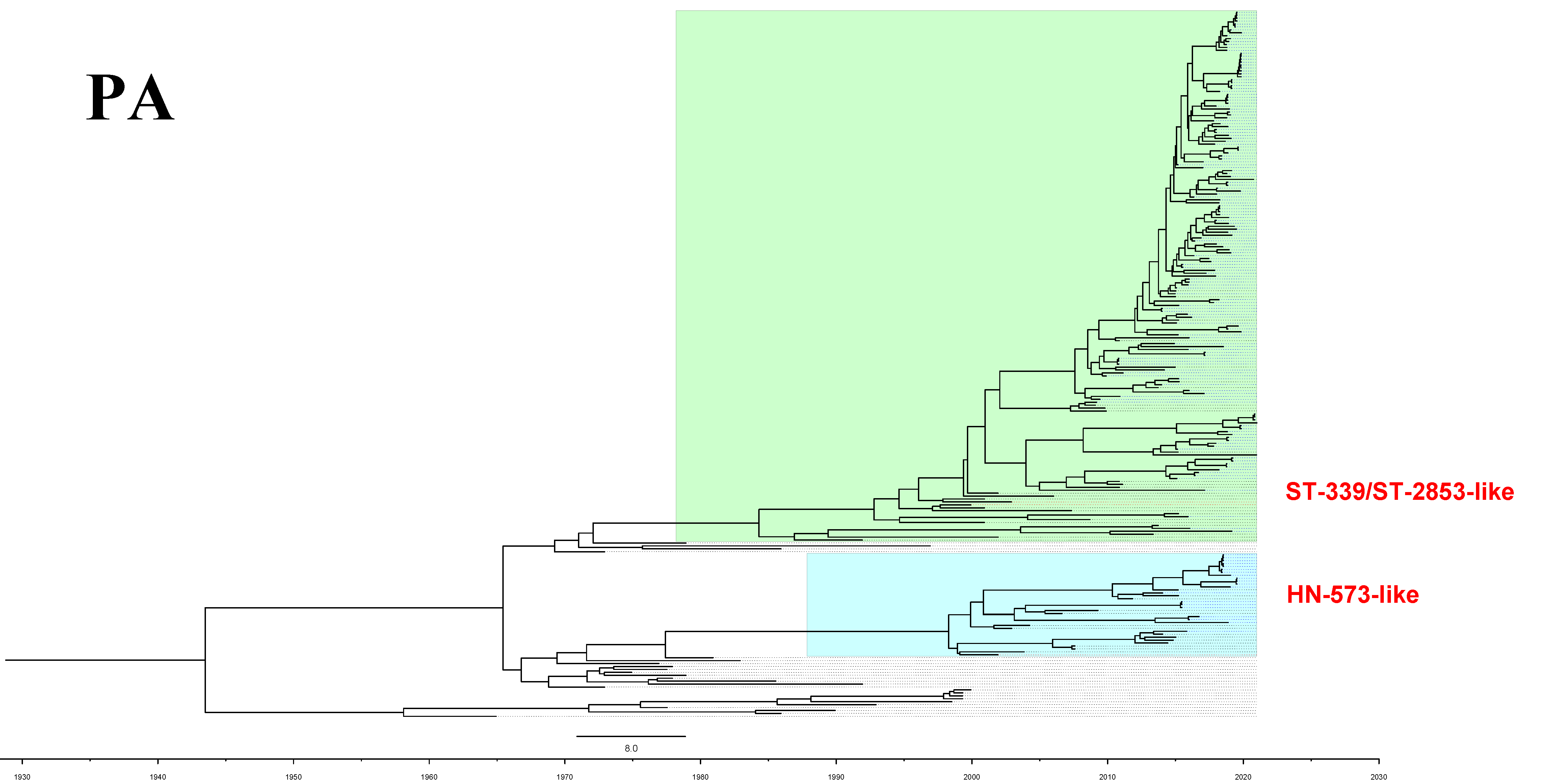

Supplement: Supplemental Material [file TEMI_A_2151380_SM4152.zip › Figure S2 _PA.tif]

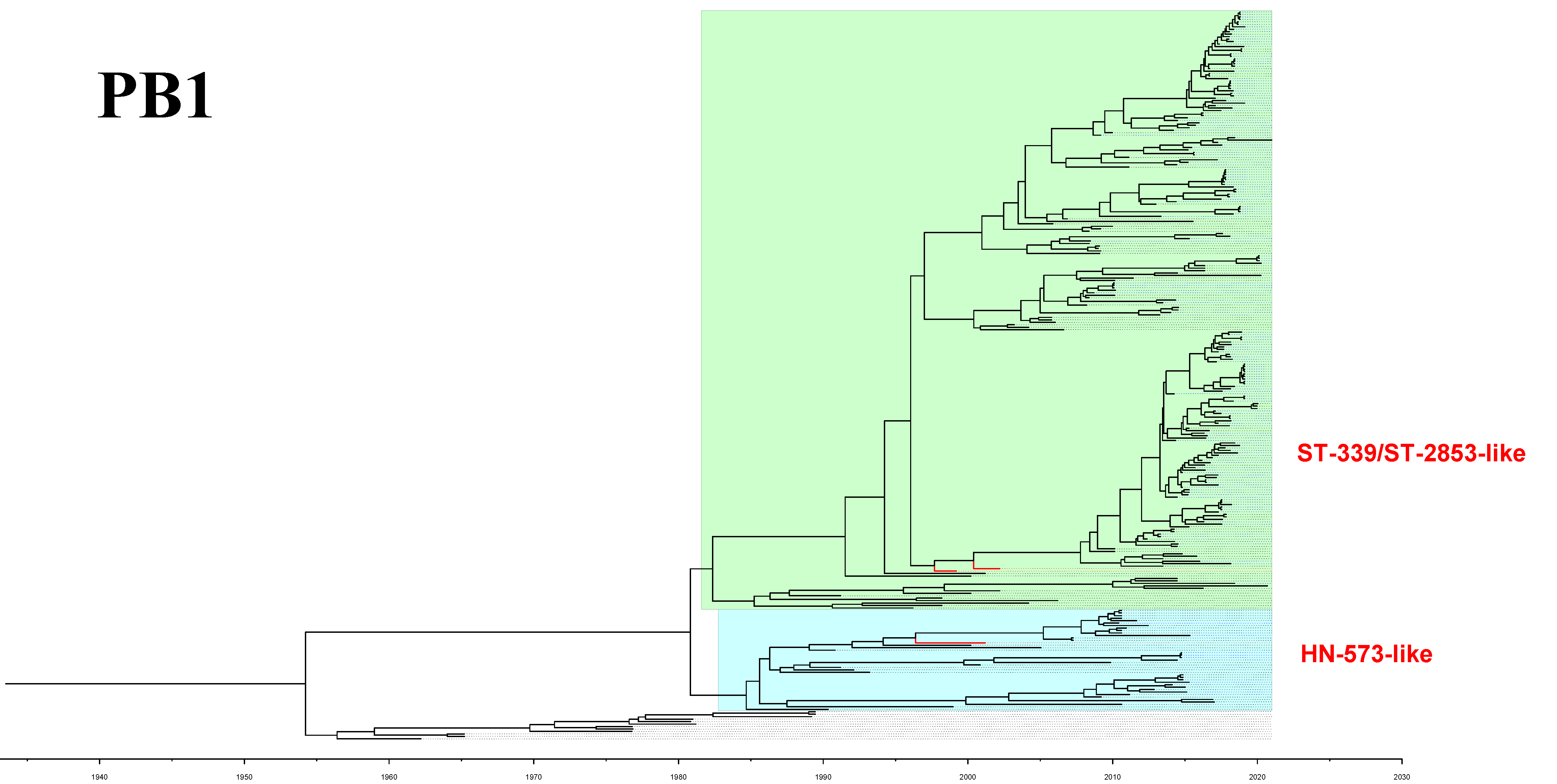

Supplement: Supplemental Material [file TEMI_A_2151380_SM4152.zip › Figure S2 _PB1.tif]

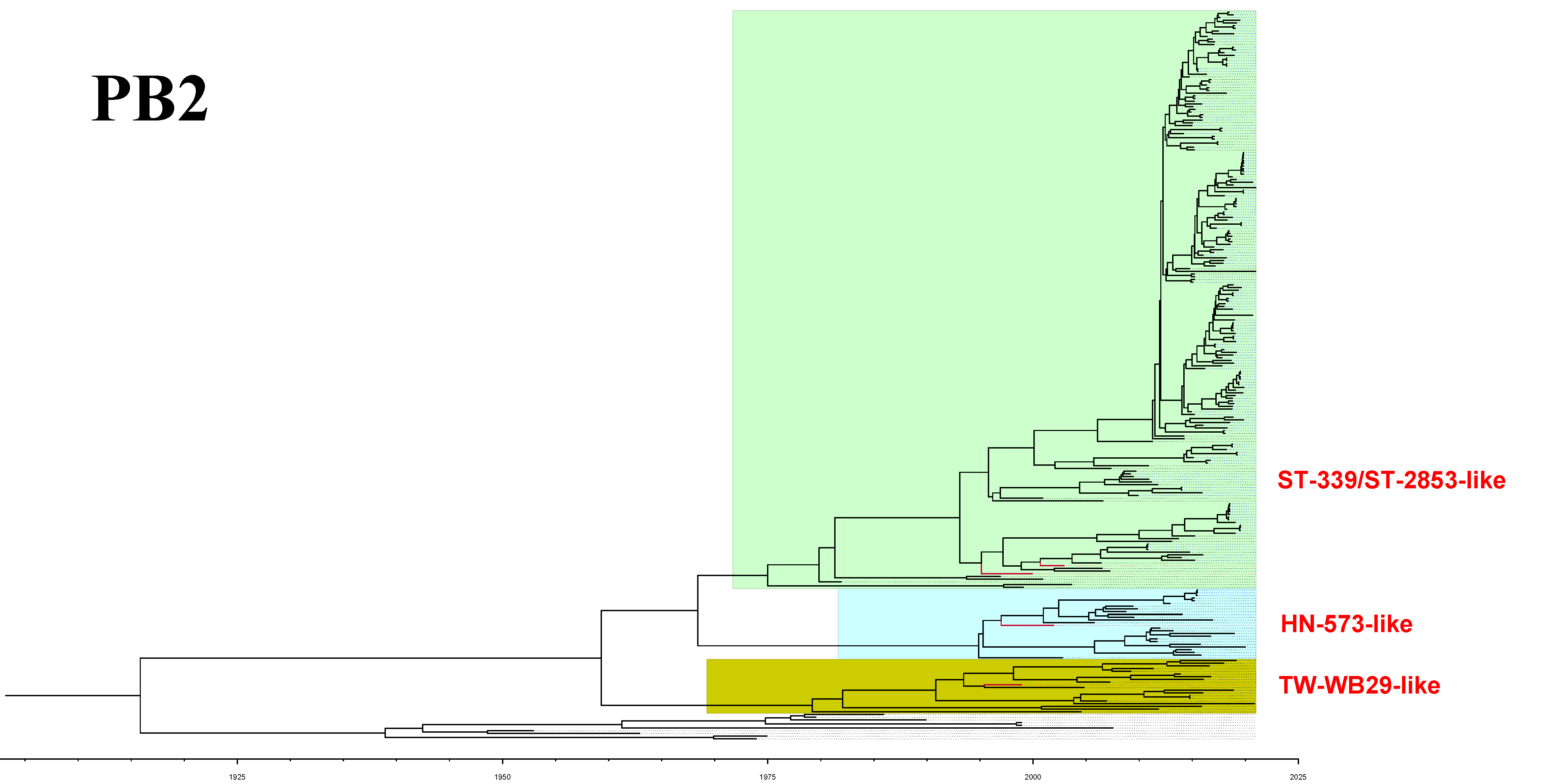

Supplement: Supplemental Material [file TEMI_A_2151380_SM4152.zip › Figure S2 _PB2.tif]

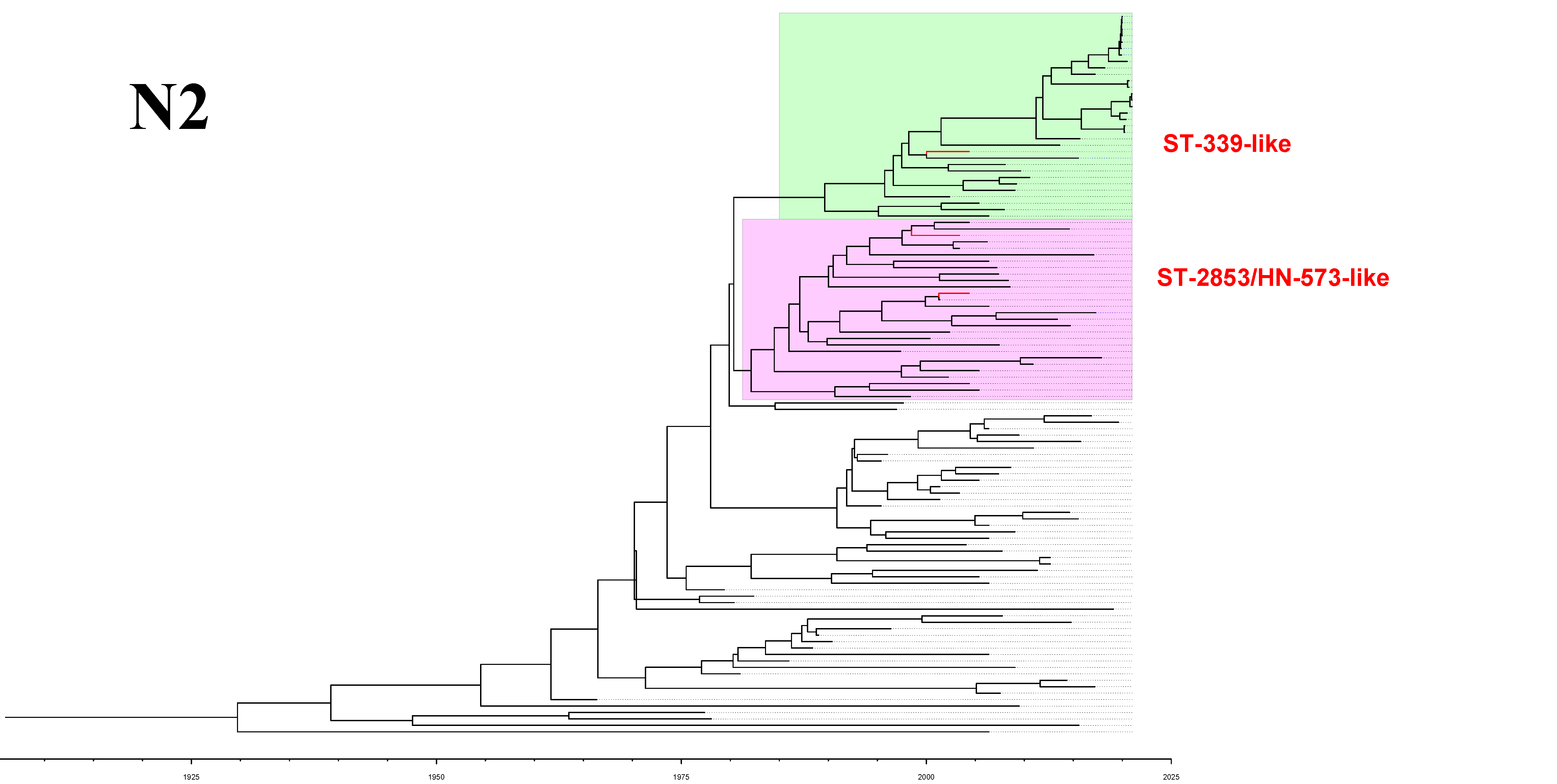

Supplement: Supplemental Material [file TEMI_A_2151380_SM4152.zip › Figure S2_N2.tiff]

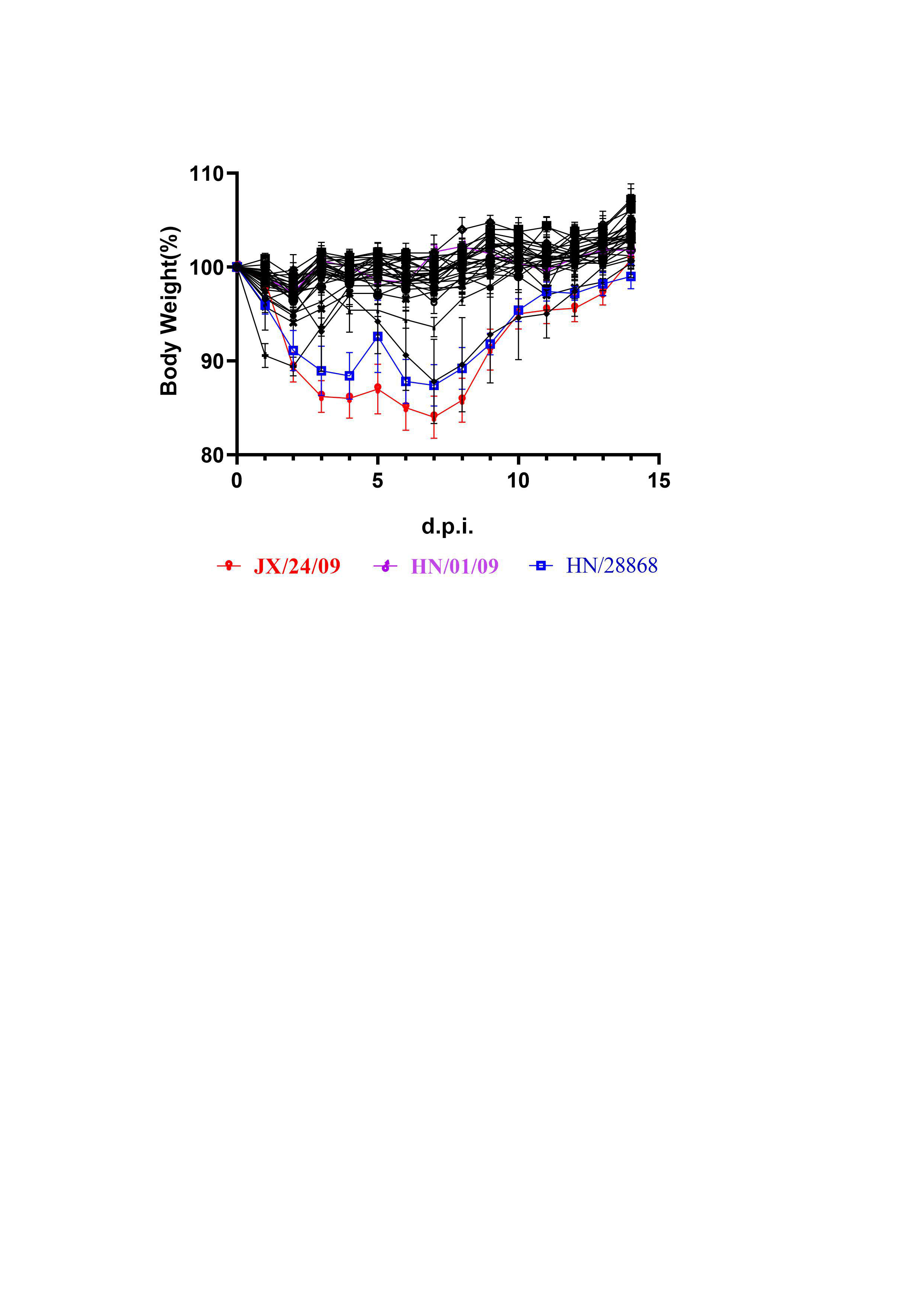

Supplement: Supplemental Material [file TEMI_A_2151380_SM4152.zip › Figure S3.tiff]

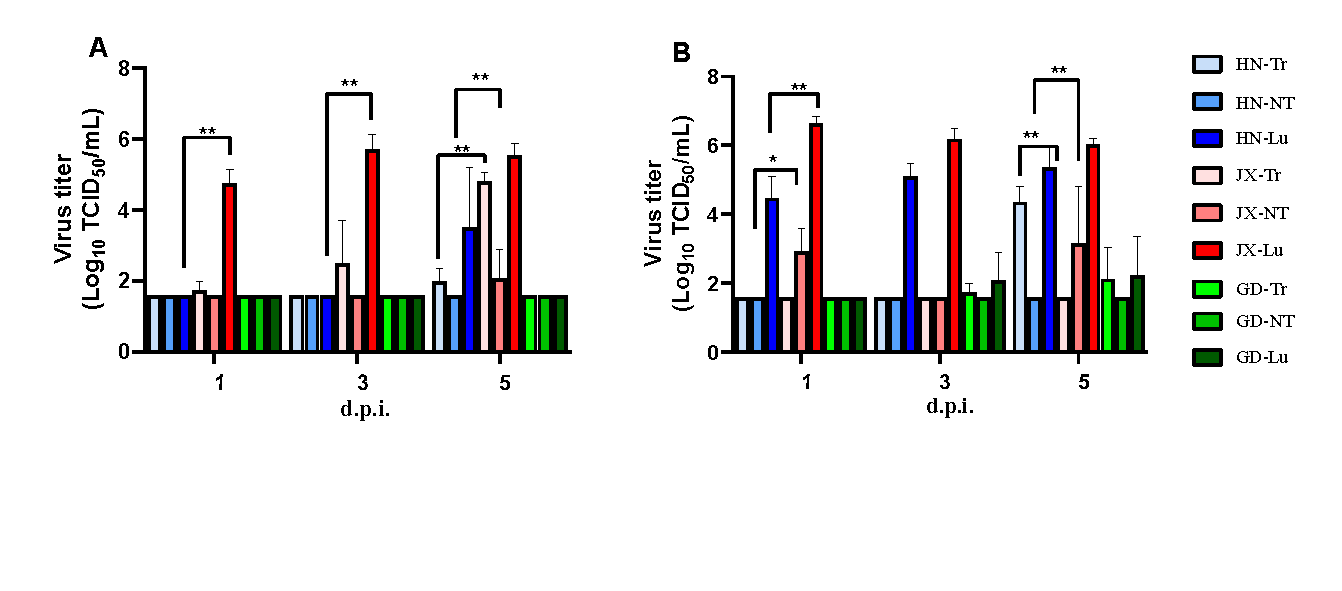

Supplement: Supplemental Material [file TEMI_A_2151380_SM4152.zip › Figure S4.tif]

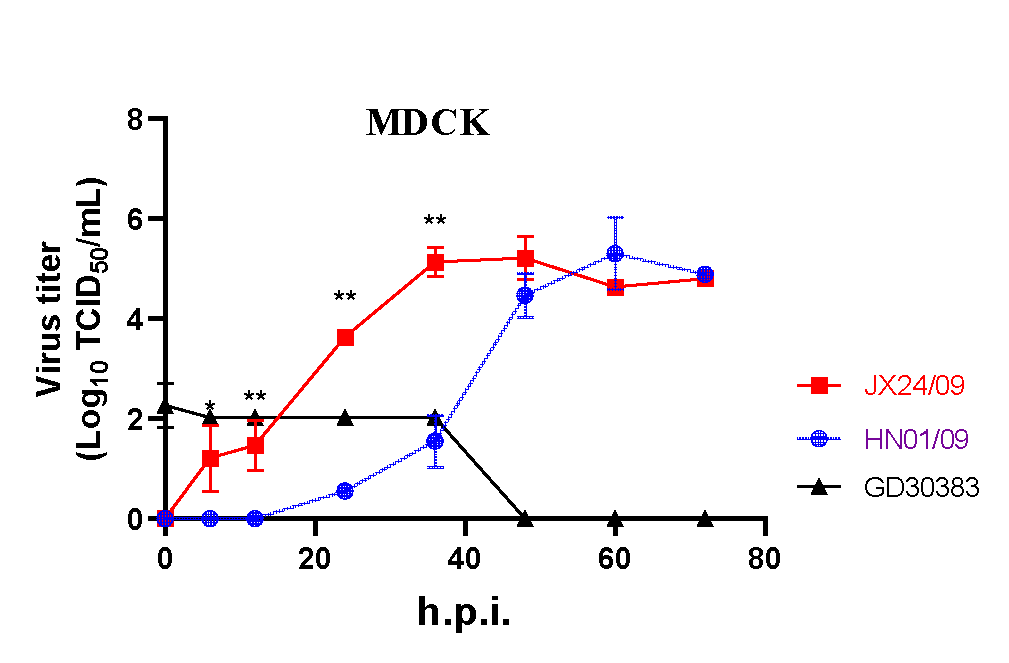

Supplement: Supplemental Material [file TEMI_A_2151380_SM4152.zip › Figure S5.tif]
